# Structural basis of diverse substrate recruitment by the HIV-1 Vpr-hijacked CRL4(VprBP) E3 ligase

**DOI:** 10.1371/journal.ppat.1014610

**Published:** 2026-10-01

**Authors:** Dianhong Wang, Weijia Ding, Jingwei Xu, Ruofan Li, Tamino Cairoli, Qi Liu, Yiying Zhu, Ye Xiang

**Affiliations:** 1 Beijing Frontier Research Center for Biological Structure, Center for Infection Biology, School of Basic Medical Sciences, Tsinghua University, Beijing, China; 2 Institute of Molecular Biology and Biophysics, ETH Zürich, Zürich, Switzerland; 3 Westlake Laboratory of Life Sciences and Biomedicine, Hangzhou, Zhejiang, China; 4 School of Life Sciences, Westlake University, Hangzhou, Zhejiang, China; 5 Cryo-EM Knowledge Hub, ETH Zürich, Zürich, Switzerland; 6 Department of Chemistry, Tsinghua University, Beijing, China; 7 Tsinghua-Peking Joint Center for Life Sciences, Beijing, China; 8 SXMU-Tsinghua Collaborative Innovation Center for Frontier Medicine, Taiyuan, China; National Institutes of Health, NIAID, UNITED STATES OF AMERICA

## Abstract

HIV-1 hijacks the host CUL4-RING E3 ligase (CRL4) complex to trigger the degradation of various restriction factors. The viral accessory protein Vpr is the key protein that recruits diverse substrates to the receptor VprBP for proteasomal degradation. However, the underlying molecular mechanism remains poorly understood. Here, we characterize the molecular architecture of the DDB1-VprBP substrate recognition unit alone and in complex with different Vpr-substrates. Our results pinpoint that the LisH domain of VprBP mediates the DDB1-VprBP dimerization. Of note, the Armadillo-like (ARM-like) domains of VprBP adopt either an “up” or “down” conformation that may assist in accommodating Vpr-substrates of divergent stoichiometries and sizes. When bound to the small Vpr substrate UNG2, the two “up” ARM-like domains wrap around and directly interact with two symmetrical UNG2 molecules. Intriguingly, the ARM-like domains adopt a “down” conformation when bound to the larger TET2 fragment. Our structures highlight that the conformational dynamics of VprBP could enable the DDB1-VprBP complex to mediate the degradation of diverse Vpr-bound host factors. These findings could facilitate the structure-guided development of CRL4(VprBP)-based targeted protein degradation.

## Author summary

The HIV-1 accessory protein Vpr promotes the proteasomal degradation of diverse host antiviral proteins via hijacking the CRL4(VprBP) E3 ligase. Moreover, investigations of the Vpr-mediated substrate recruitment characterized the Vpr protein as a natural molecular glue and a fundamental archetype for the development of proteolysis-targeting chimeras (PROTACs). Nevertheless, the mechanism by which Vpr modulates CRL4(VprBP) E3 ligase to accommodate substrates with divergent sizes and architectures is not fully understood. By determining the cryo-EM structures of DDB1-VprBP in its apo state and in complex with two Vpr-bound substrates, we unravel a profound degree of structural plasticity within the assembly, particularly involving the Armadillo-like (ARM-like) domain of VprBP, which engages in the binding of Vpr-substrates with various sizes. For small substrates, ARM-like domains adopt an “ARM-up” conformation, which cooperates with WD40 domains to create a pocket for the binding of substrates (e.g., Vpr-UNG2). Interestingly, ARM-like domain switches to an “ARM-down” conformation to avoid steric hindrance, thereby providing sufficient space for the recruitment of larger substrates (e.g., Vpr-TET2). These findings provide mechanistic insight into how DDB1-VprBP could recognize a broad range of Vpr-bound substrates and provide a molecular basis for engineering next-generation chimeric degraders.

## Introduction

The multi-subunit Cullin-RING E3 ubiquitin ligase complexes (CRLs) represent the largest E3 ligase family in mammals, comprising more than 200 members [1,2]. The core of a CRL is formed by a “C-shaped” canonical Cullin scaffold (CUL1, CUL2, CUL3, CUL4A, CUL4B, CUL5, or CUL7). The Cullin protein interacts with a catalytic RING-finger protein at its C-terminus, while its N-terminus binds to a substrate-specific recognition module. As a result, the Cullin protein positions the E2-ubiquitin in precise proximity to conjugate the substrate, thereby facilitating an efficient ubiquitin transfer [3]. Among CRLs, the CRL4 complex is involved in DNA repair and chromatin remodeling [4]. This complex consists of the scaffold proteins CUL4A or CUL4B, the RING-finger protein Rbx1, the adaptor DDB1, and one of the approximately sixty DDB1 and CUL4-associated factors (DCAFs) that function as substrate receptors [5–7].

Upon viral infection, host cells employ various factors to limit viral proliferation [8,9]. To counteract this defense, many viruses encode accessory proteins that hijack the host ubiquitination machinery, especially CRLs, to mediate the degradation of host restriction factors [10–13]. In human immunodeficiency virus 1 (HIV-1), Vpr is typically characterized as a small accessory protein of approximately 96 amino acids, with genetic diversity and length variation among different isolates and subtypes [14,15]. Vpr recruits CRL4 by binding to VprBP (also known as DCAF1), a substrate receptor protein of CRL4 [16,17]. Upon CRL4 recruitment, Vpr guides the ubiquitination and downstream degradation of many host proteins, such as UNG2, the SLX4-MUS81 complex, HLTF, TET2, MCM10, Dicer, the HUSH complex, and hHR23A [18–25]. In addition to Vpr, Vpx, a homologous protein of Vpr encoded by human immunodeficiency virus 2 (HIV-2) and specific lineages of simian immunodeficiency virus (SIV), antagonizes the dNTPase SAMHD1 by recruiting the CRL4(VprBP) ligase to trigger its degradation. Notably, certain Vpr proteins have also been reported to target SAMHD1 [26,27].

In addition to viral hijacking, CRL4(VprBP) is emerging as a formidable alternative target for proteolysis targeting chimeras (PROTACs) [28–30]. PROTACs use bivalent small molecules to bridge E3 ligases and target proteins, polyubiquitinate the protein of interest (POI), and eventually lead to its proteasomal degradation [31–33]. Many non-covalent small-molecule ligands have been developed to selectively recruit VprBP for the degradation of BRD9 and BTK [29]. Furthermore, azetidine acrylamide-derived PROTACs have been engineered to form a covalent linkage with a specific cysteine residue of VprBP, allowing for the degradation of multiple targets, such as FKBP12 and BRD4 [30]. Given that Vpr recruits the CRL4(VprBP) E3 ligase to degrade host factors, Vpr-derived peptides are also being used as chimeric recruitment motifs to induce the degradation of target proteins [34].

Despite its vital role in cellular homeostasis, viral pathogenesis and expanding PROTAC applications, a full-length structure of VprBP is still missing. While the structures of VprBP WD40 domain in complex with DDB1 provided initial insights into the assembly of CRL4(VprBP) E3 ligase [13,35], the lack of a full-length structure limits our understanding of how individual domains of VprBP cooperatively coordinate the substrate recruitment and downstream CRL4 activation. Here, we determined the structures of DDB1-VprBP in the substrate-free state and bound to two Vpr-recruited substrates. We show that the DDB1-VprBP complex forms a symmetric homodimer, in which its N-terminal Armadillo-like (ARM-like) domains adopt distinct “up” or “down” conformations. Our findings suggest that the dynamics of ARM-like domains enable the CRL4(VprBP) E3 ligase to accommodate a wide range of substrate sizes.

## Results

### DDB1-VprBP forms a homodimer in both substrate-free and Vpr-substrate-bound states

VprBP consists of 1507 amino acid residues and comprises four distinct domains: an N-terminal ARM-like domain (residues 1–817), a LisH domain (residues 818–1049), a WD40 domain (residues 1050–1389), and a C-terminal Acidic domain (residues 1390–1507) (Fig 1A) [36].

**Fig 1:**
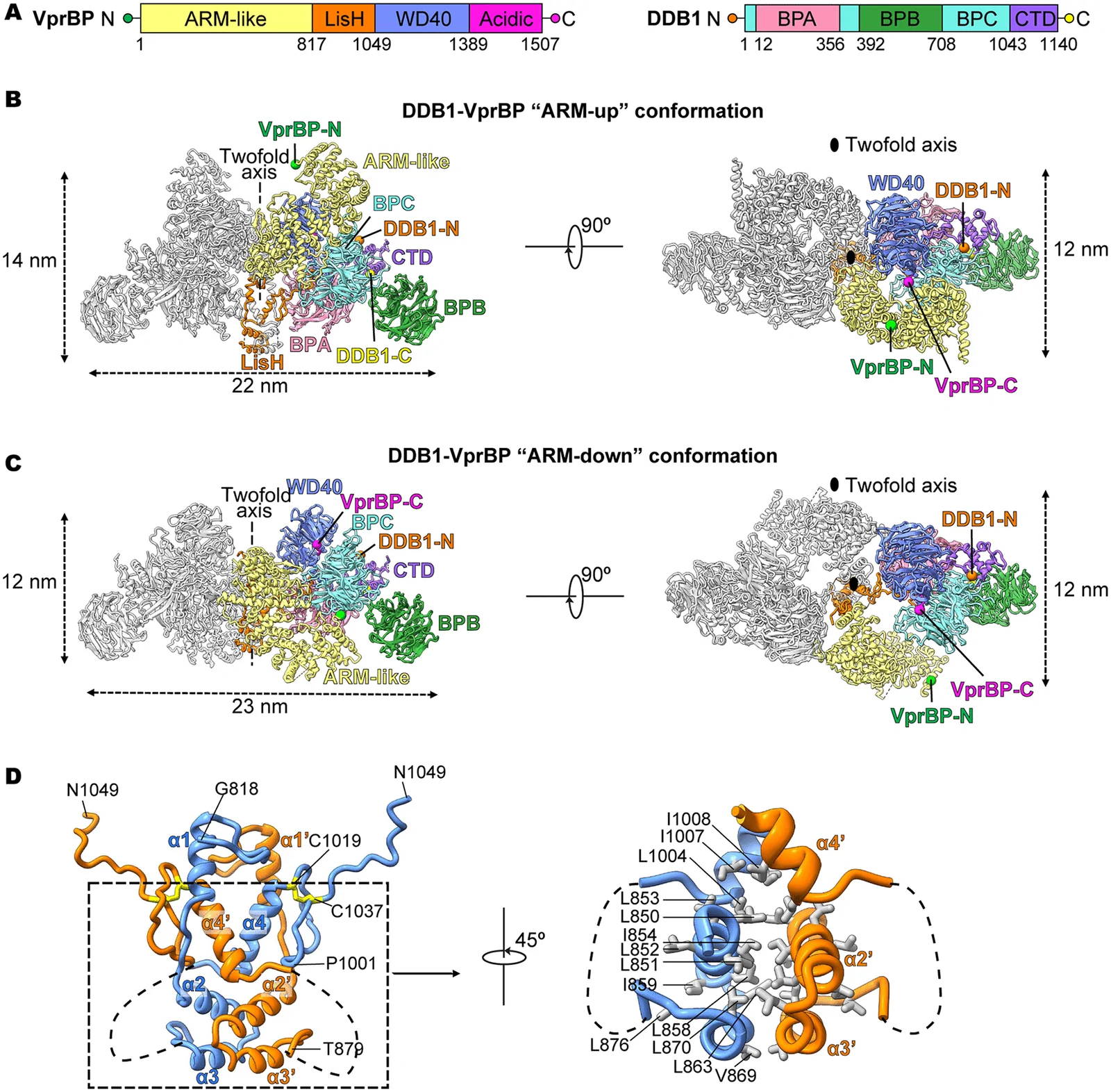
DDB1-VprBP complex assembles into a homodimer via the LisH domain. **(A)** Schematic illustration of the domain organization of VprBP and DDB1. VprBP comprises an ARM-like domain (yellow), a LisH domain (orange), a WD40 domain (blue), and a C-terminal Acidic domain (magenta). DDB1 consists of three β-propeller domains (BPA, pink; BPB, green; BPC, cyan) and a C-terminal domain (CTD, purple). **(B)** Dimeric structure of the “ARM-up” DDB1-VprBP shown as ribbons. Domains in one DDB1-VprBP protomer are color-coded according to the scheme in panel A, while the symmetry-related protomer is colored gray. **(C)** Dimeric structure of the “ARM-down” DDB1-VprBP shown as ribbons. The domains are color-coded as in panel B. **(D)** LisH domains mediate the VprBP dimerization. Left, ribbon diagrams highlighting the LisH domains (blue and orange). Disulfide bonds stabilizing the connection between loop (residues 1019-1037) and α4 are highlighted in yellow sticks. The dashed lines indicate the unresolved loop (residues 880-1000). Right, detailed view of the leucine-rich hydrophobic core at the LisH dimer interface.

To investigate how Vpr hijacks the CRL4(VprBP) E3 ligase, we first sought to determine the cryo-electron microscopy (cryo-EM) structures of DDB1-VprBP complex in the absence and presence of Vpr-substrates. We co-expressed and purified full-length DDB1 and VprBP in High Five insect cells using a baculovirus expression system. Size exclusion chromatography and SDS-PAGE analysis showed that DDB1 and VprBP formed a stable complex (S1A Fig). To obtain complexes bound with Vpr-substrates, we assembled DDB1-VprBP-Vpr-UNG2 (residues 94–313) and DDB1-VprBP-Vpr-TET2 (residues 1129–1936) complexes *in vitro*, which were further purified via GraFix [37] (S1B and S1C Fig). The peak fractions containing the complexes were pooled and applied to negative staining EM (S1D-F Fig) and cryo-EM imaging (S1G-I Fig, S1 Table).

Cryo-EM analysis revealed that the DDB1-VprBP complex forms a homodimer (S2 Fig). Intriguingly, 3D classification of the DDB1-VprBP complex revealed two distinct conformations of the ARM-like domain (hereafter referred to as “ARM-up” and “ARM-down”) (S2 Fig). Further multireference-based 3D classification analysis revealed that approximately 39% of particles were in the “ARM-up” state, while approximately 49% of particles were in the “ARM-down” state (S2 Fig). Notably, we did not observe the dimers that with one ARM-like domain in the "up" state and the other in the "down" state (S2 Fig), suggesting that the conformational transitions of the ARM-like domains within the dimer are cooperative. Regarding the Vpr-substrate-bound complexes, the DDB1-VprBP-Vpr-UNG2 complex formed a homodimer (S3 Fig). In contrast, the DDB1-VprBP-Vpr-TET2 complex exhibited asymmetric stoichiometry, with only a single copy of TET2 bound to the DDB1-VprBP-Vpr dimer (S4 Fig). Consequently, we applied different symmetries during reconstruction (C2 symmetry for DDB1-VprBP-Vpr-UNG2 and C1 for DDB1-VprBP-Vpr-TET2). Ultimately, we determined the structures of the DDB1-VprBP complex in “ARM-up” conformation, the DDB1-VprBP complex in “ARM-down” conformation, the DDB1-VprBP-Vpr-UNG2 complex, and the DDB1-VprBP-Vpr-TET2 complex at global resolutions of 4.2 Å, 9.6 Å, 3.7 Å, and 7.7 Å, respectively (S5 Fig and S6 Fig).

Atomic models of VprBP, Vpr, and DDB1 were built based on the density map of DDB1-VprBP-Vpr-UNG2, using deposited crystal structures as references (PDB ID: 5JK7) [13]. For Vpr-recruited substrates, we docked the crystal structures of UNG2 (residues 94–313, PDB ID: 5JK7) or TET2 Δ1481–1843 (residues 1129–1936 with flexible region 1481–1843 removed, PDB ID: 7NE3) [38] into the corresponding density maps and refined them as rigid bodies. Statistics for cryo-EM data collection, structure determination, model building, and model refinement are summarized in S1 Table.

Due to locally poor map quality, several regions (VprBP: residues 195–322, 695–715, 880–1000, 1315–1327, 1394–1507; Vpr: residues 80–96; DDB1: residues 1018–1022, 1117–1118) were omitted from the final model, mainly due to potential structural dynamics. Interestingly, this is consistent with the AlphaFold3-predicted structure of VprBP, showing high intrinsic disorder in these regions (S7 Fig). Moreover, these flexible regions have been reported to participate in essential cellular processes. For instance, the long loop (residues 195–322) within ARM-like domain has been previously implicated in histone H2A phosphorylation [39]. The large insertion (residues 880–1000) at LisH domain is reported to facilitate histone H3 binding [40]. Furthermore, while the C-terminal Acidic domain was not resolved, the linker extending from the WD40 domain clearly indicates that the Acidic domain is positioned within the open pocket formed between the ARM-like and WD40 domains (S8 Fig). This domain is critical for the direct interaction and recruitment of the tumor suppressor Merlin to CRL4(VprBP) E3 ligase complex for degradation [41–43].

### LisH domain mediates the VprBP dimerization

Our structure reveals that VprBP mediates the dimerization of DDB1-VprBP complex (Fig 1B and 1C), assembling into a winged-bolt-like structure in the “ARM-up” state (Fig 1B). The central stem of the complex is formed by LisH domains from each VprBP protomer. In detail, the four helices (α1–4) of one LisH domain engage in intermolecular contacts with helices from the symmetry-related subunits. The short helices of the α1-α1’ pair at the top of the stem do not interact with each other. Remarkably, the adjacent α4-α4’ helical pair interlaces to form a V-shaped structure, with their N-termini converging on the horizontal α2-α2’ pair (Fig 1D). Helices α2 and α3 form a canonical helix-turn-helix LisH motif [44], packing tightly against their symmetry-related counterparts. As a result, the α2–4 pairs of the LisH domains form a compact hydrophobic core that mediates the VprBP dimerization (Fig 1D and S9A Fig), consistent with previously characterized LisH motifs [45–47] (S9B Fig). This dimerization is further stabilized by a disulfide bond (C1019-C1037) in the loop following α4 (Fig 1D).

### ARM-like domain adopts “up” or “down” conformations

The N-terminal ARM-like domain extends outward by ~100 Å from the dimer symmetry axis (Fig 2A). Each ARM-like domain has 817 residues that form 12 armadillo-helical repeats (AHR1–12). Each repeat is composed of three α-helices arranged in a triangular shape (Fig 2B). These 12 AHRs together assemble into a right-handed supercoiled α-solenoid structure (rise = ~9 Å, twist = ~36°) (Fig 2C). The armadillo repeats pack mainly via hydrophobic interactions, whereas the solvent-exposed surface is enriched with negatively charged residues (Fig 2D).

**Fig 2:**
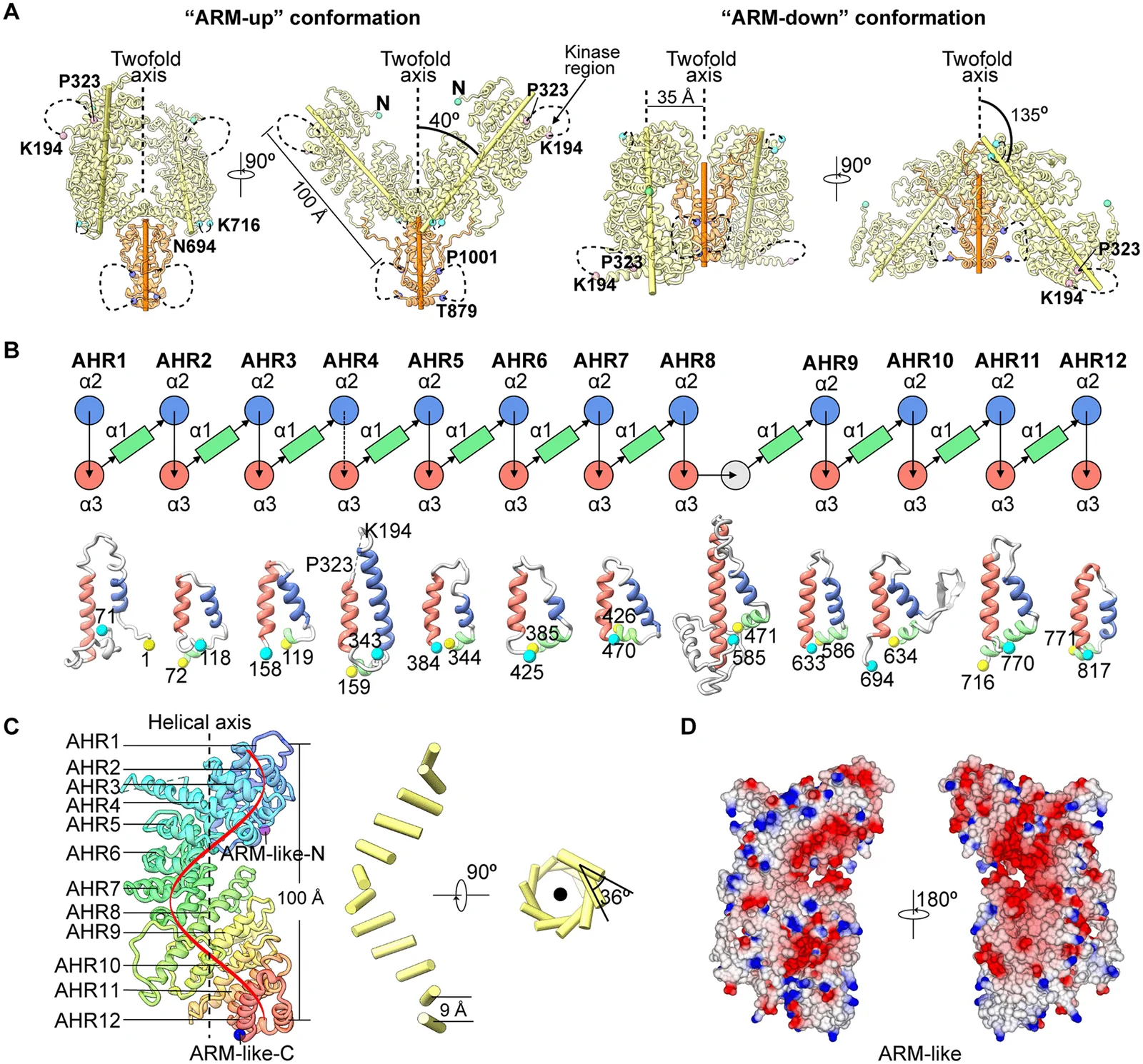
The ARM-like domain adopts “up” or “down” conformations. **(A)** Conformational states of the VprBP ARM-like domain. Ribbon representations of the “ARM-up” (left) and “ARM-down” (right) states show the spatial relationship between the ARM-like domain (yellow) and the LisH domain (orange). The dimeric twofold symmetry axis is shown to highlight the relative position of the ARM-like domains in these two states. **(B)** Topology and repeat organization of the ARM-like domain. Top, topology diagram showing the connectivity of the ARM-like domain. Bottom, individual structures of the 12 AHRs. **(C)** 12 AHRs assemble into a right-handed helical solenoid. Left, ribbon diagram of the ARM-like domain, rainbow-colored from the N-terminus (blue) to the C-terminus (red). Right, schematic diagrams showing the packing of the AHRs in the ARM-like domain. Each cylinder represents one AHR. **(D)** Electrostatic surface charge distribution of the ARM-like domain (red, negative; white, neutral; blue, positive).

We then compared the “ARM-up” and “ARM-down” states and found that the ARM-like domain exhibits a significantly different interaction profile with other domains. In the “ARM-up” conformation, α-solenoids embrace WD40 domains, forming an open pocket at the distal end of the LisH stem. In contrast to the “ARM-up” state, the ARM-like domain in the “ARM-down” conformation undergoes an approximately 95° rotation around a hinge loop (residues 817–828) that bridges the LisH and ARM-like domains (S10 Fig). The rotation of ARM-like domain cannot form the open pocket observed in the “ARM-up” state. Instead, it exposes the WD40 domains of VprBP at the distal end of the stem.

### The VprBP WD40 domain binds DDB1 that exhibits a dynamic BPB conformation

Extending from the central stem, the WD40 domain of VprBP folds into a seven-bladed β-propeller (S11A Fig), with its pseudo-sevenfold axis tilted at ~55° relative to the twofold axis of the complex (S11B Fig). In the DDB1-VprBP complex, the WD40 domain primarily mediates interactions with DDB1. In detail, the H-box of the WD40 domain (residues 1050–1079) forms a helix-turn-helix motif and inserts into the concave cleft between the BPA and BPC domains of DDB1 (Fig 3A). In addition to the WD40 domain, we observed additional interactions between DDB1 and other parts of VprBP. For instance, a loop of the C-terminal part of the LisH domain traverses the outer surface of DDB1 BPA, in which three positively charged residues (K1041, R1043, and R1044) form salt bridges with the BPA (Fig 3A). Moreover, there are additional interactions between DDB1 and the loops within the ARM-like domain of VprBP (Fig 3B).

**Fig 3:**
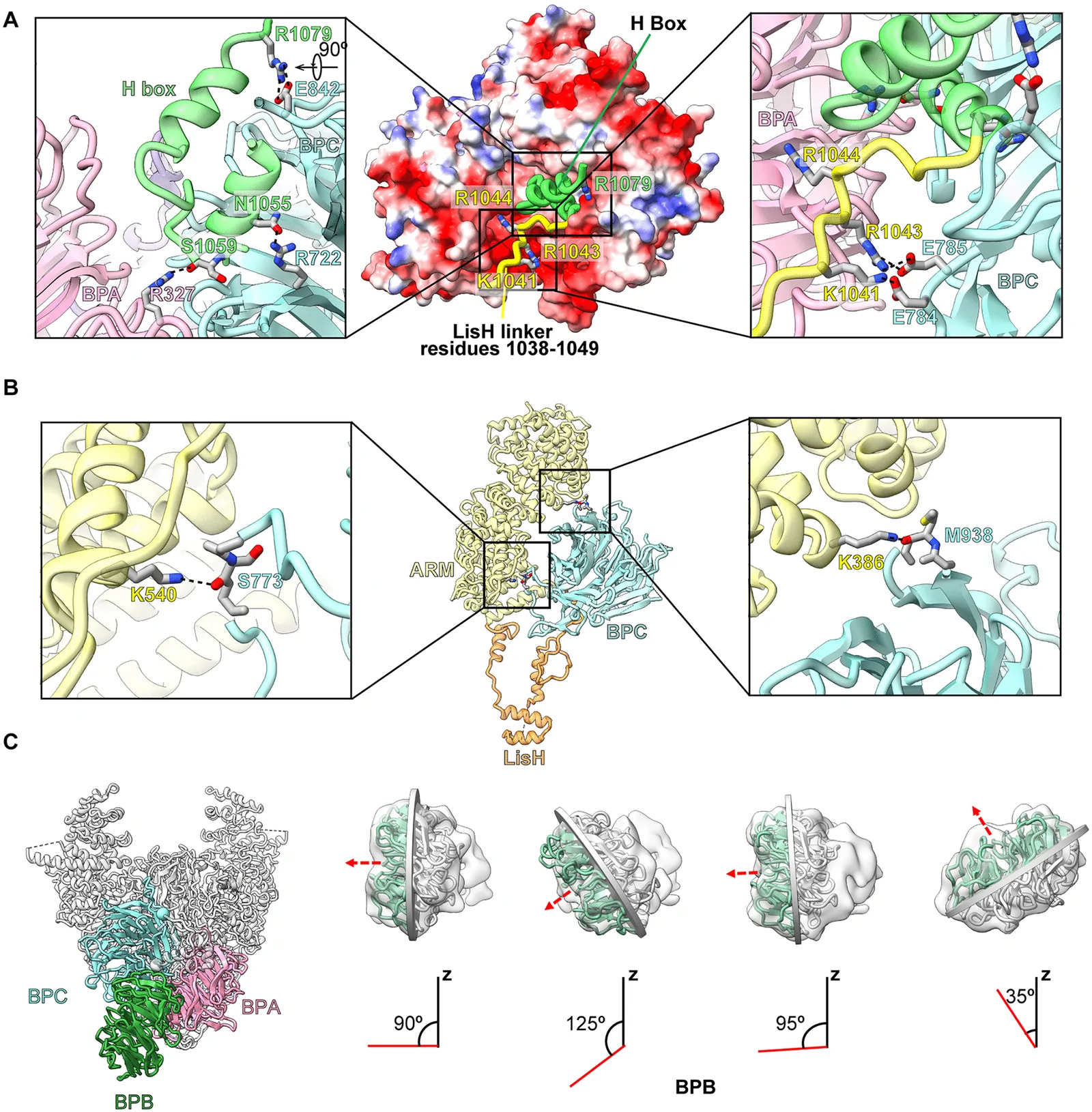
The WD40 domain of VprBP binds DDB1, which exhibits a dynamic BPB domain. **(A)** Interactions between VprBP and DDB1. The DDB1 BPA and BPC domains are shown in surface representation and colored by electrostatic potential (red, negative; white, neutral; blue, positive). The H-box of the VprBP WD40 domain (green) and the linker between LisH domain and WD40 domain (yellow) are shown as ribbons. The detailed contact interfaces are zoomed in to highlight key residues involved in the interactions. **(B)** Interactions between the VprBP ARM-like and DDB1 BPC domains. The ARM-like, LisH, and BPC domains are colored yellow, orange, and cyan, respectively. The zoomed-in views highlight the interfacial residues, which are labeled and shown as sticks. **(C)** Conformational flexibility of the DDB1 BPB domain. Left, overall structure of DDB1 (BPA, pink, BPB, green, BPC, cyan) in the DDB1-VprBP complex. Right, ribbon diagrams showing the dynamic conformations of the DDB1 BPB domain (green). The tilt angles represent the deviation of the BPB domain pseudo-sevenfold axis relative to the z axis. The cut-in plane, anchored at the centroid of the BPB domain, was generated with the first two eigenvectors of the domain. Red arrows indicate the direction of the pseudo-sevenfold axis. The ribbons localized to one side of the plane, which are involved in CUL4A recruitment, are colored light green.

Two DDB1 molecules attach to the WD40 domains of VprBP, assembling into the twofold symmetrical wing-like structure of the complex. DDB1 is a 1140 amino acid protein that has three interconnected seven-bladed β-propeller domains (BPA, BPB, and BPC), which are arranged triangularly around a short C-terminal domain (CTD) (Fig 3C). In the DDB1-VprBP complex, DDB1 adopts an overall conformation and domain organization similar to previous reports [48]. The BPA and BPC are in close proximity to each other, forming an open cavity at their interface, while two flexible linkers (residues 392–393, 708–709) connecting BPB to BPC position the BPB at the distal tip of each wing.

In contrast to the BPA and BPC, the density corresponding to the BPB is relatively weak, likely due to its conformational dynamics. To investigate this, we performed focused 3D classification on the BPB, which revealed that the BPB adopts various orientations relative to the BPA and BPC (Fig 3C). Rigorous structural analysis indicated that the BPB could rotate around the flexible linkers with an angular range from 35° to 125° (Fig 3C). In line with the rotational flexibility observed in other CRL4 E3 ligases [35,49,50], DDB1 likely utilizes the conformational dynamics to orient the CRL4 catalytic module, facilitating the ubiquitination of diverse substrates (S12 Fig).

### Vpr recruits UNG2 to the DDB1-VprBP complex in the “ARM-up” state

Previous studies have reported that Vpr can recruit a set of substrates with their size ranging from 30 to 200 kDa to the DDB1-VprBP complex through interactions with the VprBP WD40 domain [18,22,23]. Consistent with these reports, we observed Vpr-UNG2 substrate bound to the open pocket formed by the “ARM-up” ARM-like and WD40 domains in the DDB1-VprBP-Vpr-UNG2 complex (Fig 4A). Importantly, we did not observe “down” ARM-like domains in any of the DDB1-VprBP-Vpr-UNG2 complex particles.

**Fig 4:**
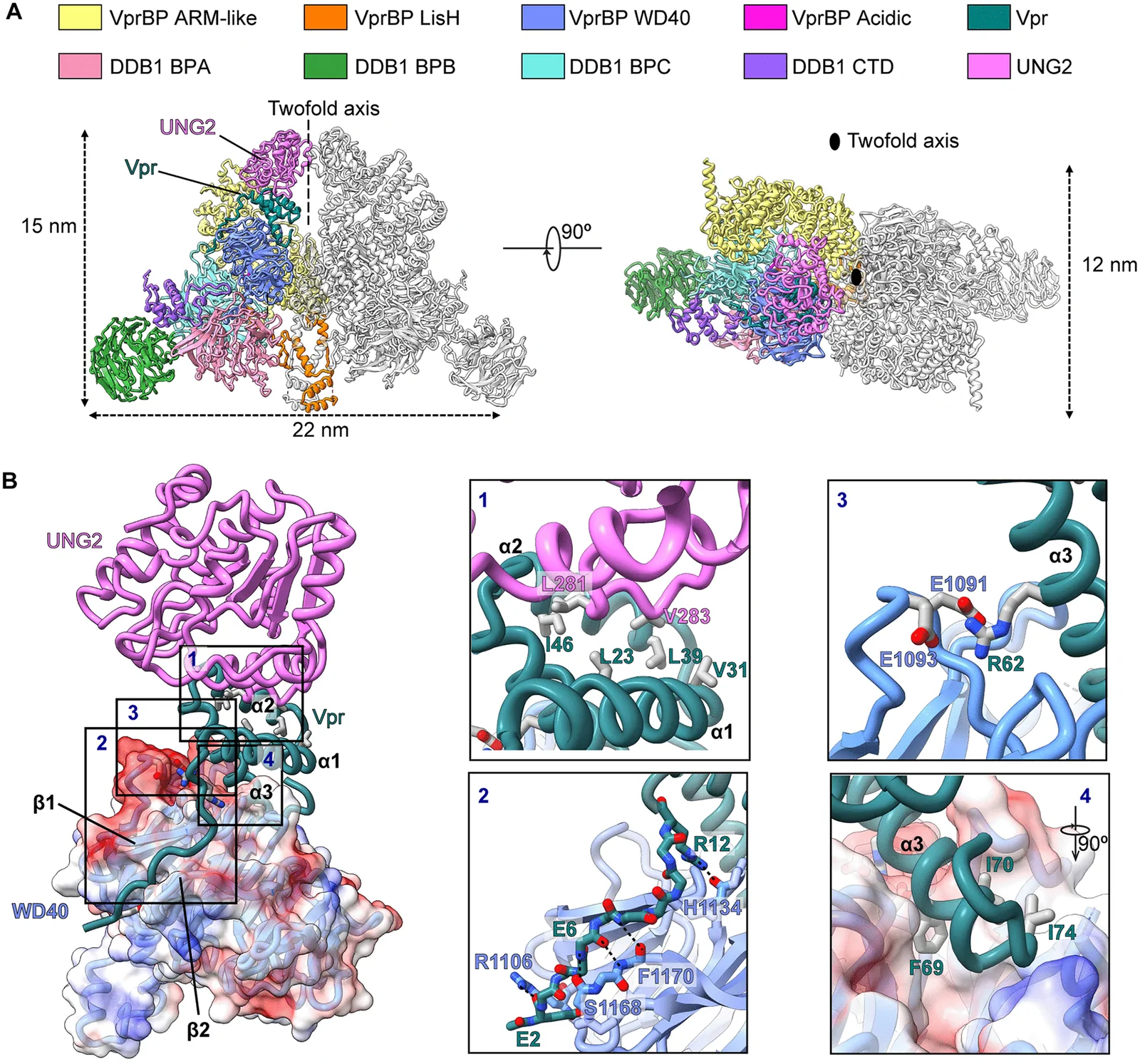
Vpr recruits two UNG2 molecules to the DDB1-VprBP complex in the “ARM-up” state. **(A)** Overall architecture of the DDB1-VprBP-Vpr-UNG2 quaternary complex. The structure is shown as ribbons. The domains are color-coded according to the scheme shown at the top. **(B)** Interactions among WD40 domain, Vpr, and UNG2. The WD40 domain is shown as a semi-transparent surface overlaid with ribbons. Vpr (dark green) and UNG2 (pink) are shown in ribbon representation. The zoomed-in views highlight the electrostatic and hydrophobic interactions between the WD40 domain and Vpr, as well as the hydrophobic interactions between Vpr and UNG2. Key residues involved in these interactions are labeled and represented as sticks.

Unlike previously reported crystal structures of the DDB1-VprBP_WD40_-Vpr-UNG2, the DDB1-VprBP-Vpr-UNG2 complex has completely different structural organization (S13 Fig). Three α helices of Vpr attach tightly to the exterior surface of WD40 domain (Fig 4B). The N-terminal region of Vpr binds to a cleft between blades β1 and β2 of WD40 domain, while the distal C-terminal region of helix α3 forms hydrophobic interactions with the central surface of the β-propeller (Fig 4B). The interaction between Vpr and VprBP is further stabilized by salt bridges between residue R62 at the N-terminus of helix α3 and an acidic loop (E1091-E1093) of the WD40 domain (Fig 4B).

The attachment of Vpr exposes the peripheral surface of helices α1 and α2, which bind to the substrate UNG2, a small substrate with a molecular weight of ~35 kDa (Fig 4A, B). In addition, we observed weak densities adjacent to helix α2 of Vpr, which likely correspond to the Vpr C-terminal region (residues 80–96) (S14A Fig). Previous studies have shown that residues H78 and R88 in the C-terminus of Vpr are essential for G2-phase cell cycle arrest [51]. This region, however, does not form direct interactions with either UNG2 or the WD40 domain in the DDB1-VprBP-Vpr-UNG2 complex. Consistent with our structural analysis, cellular degradation assays showed that the Vpr R88A mutant retained its capacity to induce UNG2 depletion to an extent comparable to wild-type Vpr (S14B and S14C Fig). We speculated that the C-terminal region of Vpr remains flexible in this state, but may become ordered upon binding to other specific host factors involved in G2-phase regulation.

The two bound UNG2 molecules are arranged in an interlaced manner within the open pocket, occupying most of the space without direct contacts between each other. Of note, residues K80 and D82 of the “ARM-up” ARM-like domain are close to the residues H124, Y125, and T126 of the bound UNG2 (S15A Fig and S2 Table), implying that these interactions might contribute to stabilizing UNG2 molecules. Consistent with our structural analysis, site-directed mutagenesis analysis showed that UNG2 mutants H124A, Y125A, and T126A rescued UNG2 from Vpr-mediated degradation when they are co-expressed in HEK293T cells (S15B and S15C Fig).

### Vpr recruits TET2 to the DDB1-VprBP complex in the “ARM-down” state

Given small molecular size of UNG2 (~35 kDa) and the observed “ARM-up” state of VprBP in the complex, we hypothesized that the limited space of the open pocket formed by the “up” ARM-like domain and the WD40 domain might only accommodate small substrates. To examine this, we investigated which conformation VprBP adopts when bound to Vpr-substrates of a larger size. We therefore chose a Vpr-mediated substrate TET2, a protein with a molecular weight of ~224 kDa. After screening various truncated constructs, we found that TET2 binds Vpr only when an unstructured region (residues 1481–1843) is present in the catalytic domain (S16 Fig).

We performed cryo-EM analysis of DDB1-VprBP-Vpr-TET2 complex and determined a structure at a moderate resolution, possibly due to its structural heterogeneity (S1 Video). Interestingly, we clearly observed an additional density in close proximity to Vpr, which corresponds to the density of the substrate TET2 (Fig 5A). To verify this, we masked out the regions corresponding to DDB1, VprBP, and Vpr, and performed a rigid-body search using the TET2 catalytic domain (PDB ID:7NE3) [38] in ChimeraX [52]. In detail, we generated 100 independent orientations and translations of the TET2 structure, followed by local optimization (fit-to-map) to identify the global cross-correlation maximum. The top-scoring model showed that the TET2 catalytic core is oriented to position its intrinsically disordered region (residues 1466–1843) close to the Vpr recruitment interface (Fig 5B), which fully supports our biochemical results (S16 Fig).

**Fig 5:**
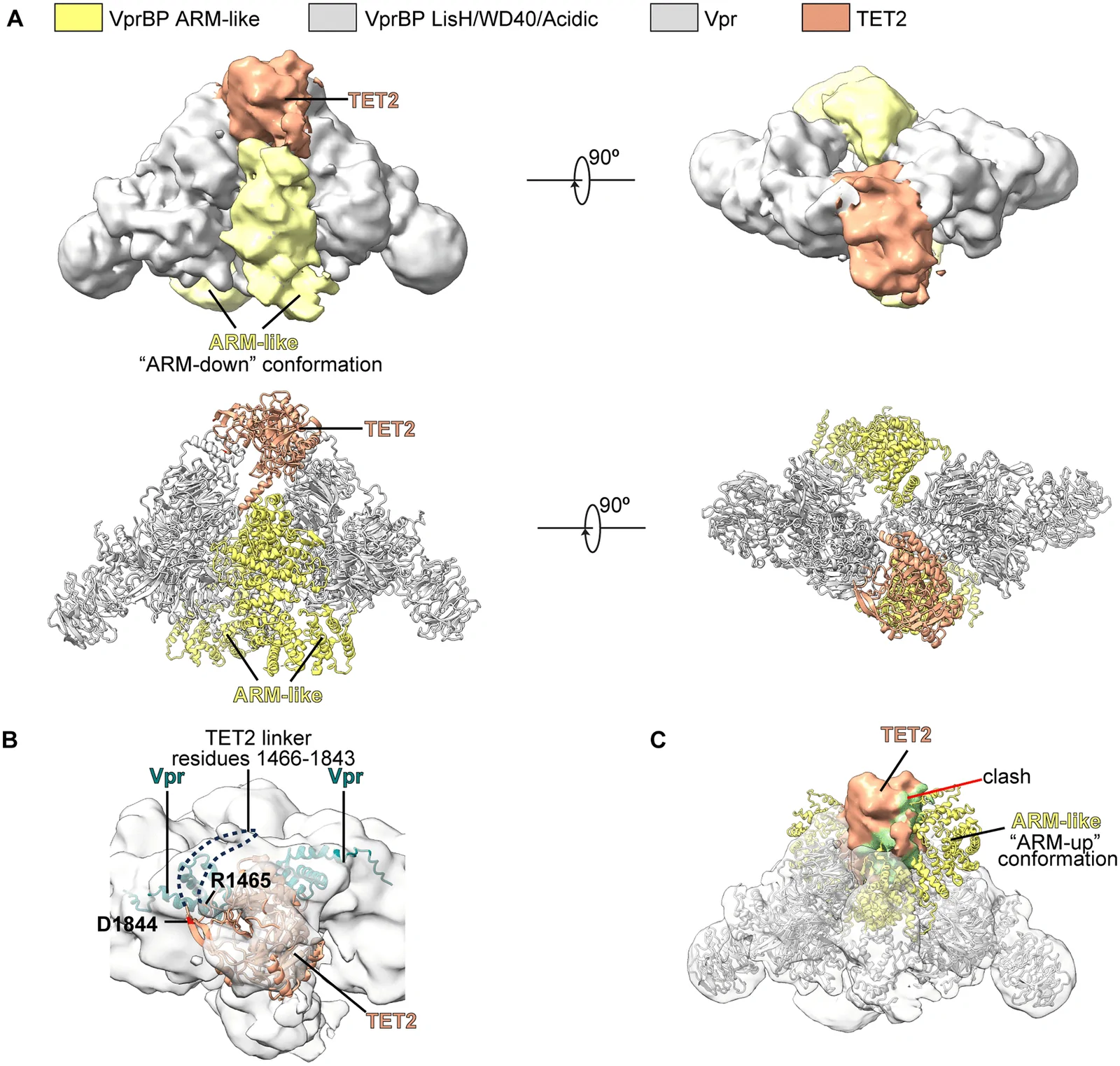
Vpr recruits a single TET2 molecule to the DDB1-VprBP complex in the “ARM-down” state. **(A)** Overall architecture of the DDB1-VprBP-Vpr-TET2 quaternary complex. The overall structure is shown in both surface (middle) and ribbon (bottom) representations. Domains are colored according to the schematic shown at the top. **(B)** Docking of the TET2 catalytic core. A detailed view illustrates the fitting of the TET2 crystal structure (PDB ID: 7NE3) into the cryo-EM density. The proximity of the TET2 internal flexible loop to the Vpr molecule validates the docking orientation. **(C)** Steric hindrance between the “ARM-up” ARM-like domain and TET2. Structural modeling reveals steric clashes between the ARM-like domain in the “ARM-up” conformation and the bound TET2. The calculated clash volume is highlighted in green, indicating that TET2 recruitment is restricted to the “ARM-down” state.

Unlike “ARM-up” state observed in the DDB1-VprBP-Vpr-UNG2 complex, our structure showed that VprBP adopts the “ARM-down” state when bound to TET2 (1129–1936), (Fig 5A and S17 Fig). The structural docking further revealed that the DDB1-VprBP-Vpr dimer binds only a single copy of TET2 on the distal surface of the complex, while the DDB1-VprBP modeled in “ARM-up” state shows severe steric clashes between the ARM-like domains and TET2 (Fig 5C). These observations suggest that VprBP must switch to the “ARM-down” conformation to accommodate large substrates like TET2.

Moreover, we performed AlphaFold prediction with two Vpr molecules and one TET2 molecule (S18A Fig) [53]. AlphaFold predicted structure revealed that residues N41, Q44 and E48 on helix α2 of Vpr might be involved in the binding of TET2 (S18A Fig). To examine this, we co-transfected cells with TET2 and either wild-type Vpr or point mutants of helix α2. Strikingly, all the mutants significantly impaired Vpr-mediated TET2 degradation, suggesting that these residues are essential for TET2 degradation (S18B and S18C Fig). In addition, we also explored the functional role of Vpr C-terminus on the TET2 degradation. Similar to UNG2, the Vpr R88A mutant could still induce TET2 degradation (S18D and S18E Fig). Taken together, Vpr recruits TET2 substrate mainly via its helix α2, whereas the flexible C-terminal region is not essential for this targeted degradation.

## Discussion

The ongoing molecular arms race between viruses and their hosts drives the evolution of intricate strategies for immune evasion and cellular control [54,55]. HIV-1 Vpr hijacks the host CRL4(VprBP) E3 ubiquitin ligase to suppress restriction factors and modulate cellular homeostasis [21,56–58]. Although several Vpr-dependent substrates have been identified, it remains unclear how Vpr manipulates the CRL4(VprBP) E3 ligase to accommodate substrates of diverse structure and size. Herein, we determined cryo-EM structures of the DDB1-VprBP complex in both substrate-free and two Vpr-substrate-bound states, providing mechanistic insights into how Vpr recruits substrates of distinct sizes and architectures to CRL4(VprBP) E3 ubiquitin ligase. By combining structural and biochemical analyses, we revealed that conformational dynamics of VprBP allow for the broad substrate recruitment.

By leveraging structural analysis, we discovered that VprBP ARM-like domain adopts two major conformations (“ARM-up” and “ARM-down”), which are coupled within the dimeric assembly (Fig 1B and 1C). In the “ARM-up” state, the ARM-like domain creates an open pocket at the distal end of the LisH stem, partially enclosing the WD40 domain, whereas the “ARM-down” conformation exposes the WD40 domain entirely. Further structural analysis of the DDB1-VprBP complex with two different Vpr-substrates indicated that these two states likely undergo substrate-dependent transitions. Binding of the small substrate UNG2 occurs in the “ARM-up” state (Fig 4A), allowing it to fit within the open pocket, while recruitment of the larger TET2 fragment requires an “ARM-down” conformation to avoid steric hindrance (Fig 5A and 5C). This conformational dynamics suggests a potential mechanism for how VprBP adapts to substrates of variable molecular size. For broad substrates of VprBP, including full-length TET2, it still requires efforts to investigate whether VprBP can use the similar conformational adaptation strategy for the binding of substrates. Moreover, recent studies have shown that VprBP can directly bind to many substrates independently of Vpr via its N-terminal ARM-like domains [59,60]. Given that the exposed surfaces differ significantly between the “up” and “down” conformations, these alternative binding sites on the ARM-like domain may help orient substrates relative to the catalytic core for the ubiquitin transfer. Nevertheless, the link between the dynamics of ARM-like domain and the efficiency of the ubiquitin transfer remains to be experimentally demonstrated.

Previous studies showed that the WD40 domain of VprBP alone is sufficient to mediate the ubiquitination of UNG2. However, the WD40 domain mediated ubiquitination is less efficient compared to that of the full-length protein [61]. Here, we observed that DDB1-VprBP forms a homodimer mainly via LisH domain. This dimerization likely increases substrate recruitment efficiency compared to the monomeric complex. Moreover, CRL4(VprBP) E3 ligase has been reported to form higher-order assembly (e.g., tetramer), which regulates substrate recruitment and ubiquitination activity [62]. Notably, previous reports of dimeric and tetrameric assemblies showed the ARM-like domain adopts “ARM-down” conformation, which is consistent with the “ARM-down” state in our cryo-EM reconstructions. It therefore suggests that structural flexibility as well as transitions in oligomeric state both contribute to its functional diversity.

In addition to the dynamics of VprBP itself, flexibility within DDB1 also contributes to structural adaptability of the CRL4(VprBP) E3 ligase. Our cryo-EM maps reveal that the BPB domain of DDB1 undergoes substantial rotational movement relative to the BPA and BPC domains (Fig 3C), consistent with recent studies [35,49,63]. Such conformational variability likely positions the CUL4 scaffold and its Rbx1–E2 partner for optimal alignment with substrates, thereby facilitating efficient ubiquitin transfer across substrates of different sizes and orientations.

Vpr plays a critical role in the HIV-1 life cycle, particularly in arresting host cells at the G2 phase to facilitate the efficient viral replication. Although several potential host factors have been identified, none of these host factors were characterized to be essential for Vpr-mediated G2 phase arrest [18,20–22]. Mutagenesis studies on Vpr have shown that arginine residues within its C-terminal region are vital for G2 phase arrest [64,65]. Our DDB1-VprBP-Vpr-UNG2 structure revealed additional weak densities near the helix α2 of Vpr, likely corresponding to its flexible C-terminal tail (S14A Fig). This suggests that the contacts between the C-terminal tail and helix α2 could constitute a putative substrate binding surface for the elusive G2 arrest factor.

The broad tissue expression and prominent roles of VprBP in various cancers make the CRL4(VprBP) E3 ligase an attractive target for PROTAC-mediated protein degradation. Recent studies have identified small molecule ligands that successfully engage VprBP [29,30,66]. As a natural molecular glue, Vpr-derived peptides offer a distinct advantage as a potent E3 ligase recruiter due to their high binding affinity and well-defined interaction interface with the DDB1-VprBP complex. This approach has recently been validated by the development of chimeric degraders utilizing Vpr-derived peptides and the BRD4 inhibitor, which successfully achieved targeted degradation of BRD4 [34]. Consistently, our structural findings elucidate the mechanism by which Vpr recruits a diverse array of substrates characterized by varying sizes and architectures. The conformational flexibility of VprBP ARM-like domain expands the accessible substrate binding capacity, providing a strategic architectural advantage for the design of next-generation PROTACs. These findings highlight the potential of leveraging virus-host interactions to expand the current strategy of E3 ligase recruiters for therapeutic applications.

## Materials and methods

### Gene cloning and protein expression

Human genes encoding DDB1, VprBP, UNG2, and TET2 were amplified by PCR from commercial human tissue cDNA libraries. The HIV-1 *Vpr* gene was cloned from a cDNA plasmid of HIV-1 NL4–3 (GenBank accession number: AF324493). The *DDB1* and *VprBP* genes were individually cloned into pFastBac1 vector at the BamHI restriction enzyme site using In-Fusion cloning kit (Takara Bio). These constructs were designed to include an N-terminal 10 × His-3 × Flag-TEV tag to the recombinant proteins. The plasmids were then transformed into *E. coli* DH10Bac to prepare the recombinant bacmids. To generate baculoviruses, purified recombinant bacmids were transfected into Sf9 cells using Cellfectin II reagent (Invitrogen, Cat. No. 2228190). Viruses harvested from two consecutive rounds of infection were used to infect High Five insect cells at a density of 2 × 10^6^ cells/mL.

A truncated fragment of human *UNG2* gene (residues 94–313) was cloned into the pET-30b vector using NdeI and XhoI restriction enzyme sites, introducing an N-terminal 6 × His-SUMO tag to the recombinant protein. Full-length *Vpr* gene was cloned into pET-30b vector using the same restriction enzyme sites, incorporating an N-terminal NusA tag to Vpr followed by an HRV3C protease cleavage site (NusA-H3C-Vpr). UNG2 and Vpr proteins were expressed in *E. coli* Rosetta strain following standard the IPTG-induced protein expression protocol (see the Novagen pET system handbook).

Truncated fragments of *TET2* gene with different lengths were cloned into the pCMV vector using XbaI and BamHI restriction enzyme sites, introducing a C-terminal twin-Strep II tag to the recombinant proteins. The TET2 constructs were transiently transfected into HEK293F cells using polyethyleneimine (PEI). The HEK293F cells were cultured in SMM 293-TII medium supplemented with 0.5% Fetal Bovine Serum (FBS).

### Purification of the DDB1-VprBP complex

DDB1 and VprBP were co-expressed in High Five insect cells. The cells were co-infected with recombinant baculoviruses encoding DDB1 and VprBP at a volume ratio of 1:1. At 48 h post-infection, the cells were harvested by centrifugation, and the cell pellets were resuspended in lysis buffer containing 20 mM HEPES (pH 7.5), 150 mM NaCl and protease inhibitor cocktail (Roche, Cat. No. 04693159001). Cells were lysed using a mixer mill (Retsch MM400), and the insoluble cell debris was removed by centrifugation. The supernatants were subjected to cobalt-charged BD TALON resin for affinity purification. After washing, the protein was eluted with imidazole. The eluate was then concentrated and further purified by size-exclusion chromatography using a Superose 6 column (Cytiva), equilibrated in a buffer containing 20 mM HEPES (pH 7.5) and 150 mM NaCl. Peak fractions corresponding to the DDB1-VprBP complex were identified by SDS-PAGE, pooled and concentrated for GraFix and cryo-EM grid preparation.

### Assembly of DDB1-VprBP-Vpr-UNG2 and DDB1-VprBP-Vpr-TET2 complexes

To obtain the DDB1-VprBP-Vpr-UNG2 complex, purified DDB1-VprBP and purified Vpr-UNG2 (residues 94–313) were assembled in vitro. For preparation of the Vpr-UNG2 complex, *E. coli* cells expressing NusA-H3C-Vpr and SUMO-UNG2(94–313) were mixed, harvested and resuspended in lysis buffer containing 20 mM HEPES (pH 7.5) and 150 mM NaCl. The resuspended cells were lysed by sonication and subsequent centrifugation. The supernatant was subjected to affinity purification using the cobalt-charged BD TALON resin. The eluate was incubated with Ulp1 protease to remove the SUMO tag. The cleaved SUMO tag was removed from NusA-H3C-Vpr-UNG2(94–313) using a Superdex 200 column (Cytiva). Subsequently, the NusA tag was removed using HRV3C protease, and the tag-free Vpr-UNG2 complex was further purified by size-exclusion chromatography on a Superdex 200 column. The purified DDB1-VprBP complex and Vpr-UNG2 complex were mixed at a molar ratio of 1:3. Excess Vpr-UNG2 complex was removed by size-exclusion chromatography on a Superose 6 column equilibrated in a buffer containing 20 mM HEPES (pH 7.5) and 150 mM NaCl. Fractions containing DDB1-VprBP-Vpr-UNG2 quaternary complex were collected and used for GraFix and cryo-EM grid preparation.

To assemble the DDB1-VprBP-Vpr-TET2(1129–1936) complex, the DDB1-VprBP-Vpr complex was first purified and then incubated with purified TET2(1129–1936). The cell lysate from High Five cells expressing DDB1 and VprBP was mixed with the bacterial lysate of NusA-H3C-Vpr and applied to cobalt-charged BD TALON resin. After HRV3C protease cleavage, the proteins were applied to a Superose 6 column to isolate the DDB1-VprBP-Vpr complex. Separately, TET2(1129–1936) was expressed in HEK293F cells and harvested 48 h post-transfection. After sonication and centrifugation, TET2(1129–1936) in the supernatant was purified using Strep-Tactin Superflow resin, followed by size-exclusion chromatography on a Superose 6 column with a buffer containing 20 mM HEPES (pH 7.5) and 150 mM NaCl. TET2(1129–1936) eluted at approximately 15.7 mL. Finally, DDB1-VprBP-Vpr and TET2(1129–1936) were mixed at a molar ratio of 1:2 and incubated at 4 °C for 2 h, and then subjected to a Superose 6 column equilibrated in a buffer containing 20mM HEPES (pH 7.5) and 150mM NaCl. Fractions containing all the four components were collected for GraFix and cryo-EM analysis.

### GraFix cross-linking

To stabilize the assembled complexes for cryo-EM analysis, purified DDB1-VprBP, DDB1-VprBP-Vpr-UNG2(94–313) and DDB1-VprBP-Vpr-TET2(1129–1936) were concentrated and cross-linked by GraFix method with a continuous glycerol and glutaraldehyde gradient [37]. Briefly, samples were loaded onto a 5 mL continuous gradient, consisting of 10–35% (v/v) glycerol and 0-0.25% (v/v) glutaraldehyde in a buffer containing 20 mM HEPES (pH 7.5) and 150 mM NaCl. Ultracentrifugation was performed at 200,000 × g using an SW 55 Ti rotor (Beckman Coulter) at 4°C for 12 h. After centrifugation, the gradient was fractionated from the bottom of the tube into 14 fractions (~ 0.4 mL each). The cross-linking reactions were quenched by adding Tris-HCl (pH 7.5) to a final concentration of 50 mM. Fractions containing the target complexes were pooled and concentrated using a 100 kDa molecular weight cut-off centrifugal filter unit (Millipore). During concentration, glycerol was removed, and the sample was exchanged to a buffer containing 20 mM HEPES (pH 7.5) and 150 mM NaCl.

### Cryo-EM specimen preparation and data collection

The cryo-EM grids were prepared using an FEI Vitrobot Mark IV. Briefly, 4 μL aliquots of the purified complex at a concentration of 0.4 mg/mL were applied to a glow-discharged lacey carbon grid (Ted Pella, Inc., Cat. No. 140916). The grids were blotted with filter paper for 12.5 s at 100% humidity and were then plunged into liquid ethane cooled by liquid nitrogen.

Cryo-EM datasets of the DDB1-VprBP complex were collected on an FEI Titan Krios electron microscope operating at 300 kV and equipped with a K2 Summit camera, a Gatan GIF Quantum energy filter and a spherical aberration (Cs) corrector. The movies were collected at a nominal magnification of 105,000 × , resulting in a pixel size of 1.091 Å/pixel using AutoMation2 software (developed by Jianlin Lei). The exposure time was 5.6 s per movie. The total accumulated dose was approximately 50 e^-^/Å^2^ distributed over 32 frames. A total of 2,194 micrographs were collected.

The datasets of DDB1-VprBP-Vpr-UNG2 were collected on an FEI Titan Krios electron microscope operating at 300 kV with a K3 Summit camera and a Gatan GIF Quantum energy filter at a nominal magnification of 81,000 × , yielding a pixel size of 1.0742 Å/pixel. A total of 3,995 movie stacks were collected. Each movie stack consisted of 32 frames acquired with exposure time of 8 s. The total accumulated dose was approximately 50 e^-^/Å^2^.

The datasets of DDB1-VprBP-Vpr-TET2 were collected on an FEI Titan Krios electron microscope operating at 300 kV with a Gatan K3 Summit camera at a nominal magnification of 22,500 × , which yields a pixel size of 1.25 Å. A total of 4,903 movie stacks were collected. Each movie stack consisted of 32 frames acquired with exposure time of 3.84 s. The total dose was around 50 e^-^/Å^2^. Detailed data collection parameters are listed in S1 Table.

### Cryo-EM imaging processing

The collected movie stacks were aligned, dose-weighted and summed using MotionCor2 [67]. Contrast transfer function (CTF) parameters for each micrograph were estimated using Gctf [68]. Particles classification and reconstruction were performed in RELION 3.0 [69] and cryoSPARC 5.0.0 [70] The detailed data processing workflows are summarized in S2-S4 Fig.

For the DDB1-VprBP dataset, a total of 564,312 particles were picked and extracted using Gautomatch (http://www.mrc-lmb.cam.ac.uk/kzhang/Gautomatch/). After several rounds of reference-free 2D classification, 369,342 particles exhibiting well-defined structural features were selected and subjected to 3D classification with C2 symmetry imposed, using a spherical model as the initial reference. Particles were accumulated largely in two classes, of which the reconstructed maps have obviously different conformations (“ARM-up” conformation and “ARM-down” conformation). To separate the particles from these two conformations, particles selected from the 2D classification were subjected to multi-reference 3D classification using the two conformational maps as initial references. The 3D classification results showed that 39% of particles were classified into “ARM-up” conformation and 49% of particles were classified into “ARM-down” conformation. The particles from each conformation were selected separately and subjected to refinement. The final reconstructions resulted in a map at a resolution of 4.2 Å for the “ARM-up” conformation and a map at a resolution of 9.6 Å for the “ARM-down” conformation.

For the DDB1-VprBP-Vpr-UNG2 dataset, a total of 1,601,375 particles were picked and extracted using Gautomatch. 764,127 particles were selected after several rounds of 2D classification. Further 3D classification with C2 symmetry imposed revealed a dominant class resembling “ARM-up” state. After further 3D classification, 145,568 particles were selected for 3D refinement and local CTF refinement, yielding a reconstruction at a resolution of 3.7 Å. To further improve the map quality, particles of the DDB1-VprBP-Vpr-UNG2 protomer were re-centered and re-extracted. The extracted particles were subjected to 3D classification without pose alignment. The particles from the best-resolved class were selected and used for 3D refinement, yielding a map of DDB1-VprBP-Vpr-UNG2 protomer at a resolution of 3.6 Å.

For the DDB1-VprBP-Vpr-TET2 complex dataset, the particles were first picked using Blob picker from 4,903 micrographs and extracted at a binning factor of 4. After 2 rounds of 2D classification, high-quality particles were selected and used for Topaz training [71]. A total of 1,410,460 predicted particles were extracted at a binning factor of 4. These particles were subjected to one round of 2D classification, from which 300,104 good particles were strictly selected for *ab initio* reconstruction to generate initial 3D references. To recover potential good particles, a broader pool of 1,215,705 less-strictly selected particles was subjected to heterogeneous refinement with C1 symmetry imposed using one good class and four bad classes derived from the *ab initio* models. 579,527 particles were selected and re-extracted at a binning factor of 2. These particles were then subjected to multiple successive iterations of NU-Refine and 3D classification to further resolve conformational and compositional heterogeneity. Finally, 144,145 particles were reconstructed to an overall resolution of 7.7 Å without imposing any symmetry.

Resolutions of all the reconstructed maps were estimated based on the Fourier shell correlation (FSC) between two half maps based on the 0.143 FSC criterion (S6 Fig). Local resolution maps were estimated using the local resolution module in RELION 3.0 and were displayed using UCSF ChimeraX (S6 Fig) [52].

### Structural modeling and validation

The cryo-EM density map of DDB1-VprBP-Vpr-UNG2 was sharpened using the software DeepEMhancer [72]. The crystal structure of DDB1-VprBP_WD40_-Vpr-UNG2 (PDB ID: 5JK7) was docked into the sharpened map as an initial model using fit_in_map function in ChimeraX. The VprBP ARM-like domain was modeled using Robetta (https://robetta.bakerlab.org). The LisH domain was manually built in COOT [73]. The individual models were assembled into a composite model and manually adjusted in COOT. The composite model was then subjected to iterative cycles of real-space refinements and manual adjustments against the density map using RosettaCM [74], *phenix.real_space_refine* in PHENIX [75], and COOT.

Atomic model of DDB1-VprBP “ARM-up” conformation was generated by fitting the DDB1-VprBP coordinates from DDB1-VprBP-Vpr-UNG2 model into the corresponding density, followed by real-space refinement using *phenix.real_space_refine*. The DDB1-VprBP “ARM-down” conformation model was built by docking of the individual domain models derived from the high-resolution structures into the density map using *phenix.dock_in_map*. The docked domains were then refined as rigid bodies using *phenix.real_space_refine*.

The structure of DDB1-VprBP-Vpr-TET2 was built using similar rigid body fitting and refinement strategy. The crystal structure of TET2 fragment (PDB ID: 7NE3) was used for the docking. Model quality was assessed using Molprobity scores and Ramachandran plots (S1 Table).

### Vpr-mediated degradation of TET2 and UNG2 in cell

The cDNA fragments encoding human TET2 (GenBank accession number: NM_001127208.3) and UNG2 (GenBank accession number: NM_080911.3) were PCR-amplified from a human cDNA library. The sequences corresponding to residues 1129–1936 of TET2 and full-length UNG2 were cloned into the pCMV vector to generate constructs with a C-terminal Strep tag. Three mutants of UNG2 (H124A, Y125A, and T126A) were generated by site-directed mutagenesis. The gene encoding HIV-1 Vpr was cloned into the vector pCMV to produce an N-terminal Flag-tagged fusion protein. Site-directed mutagenesis of Vpr was performed by PCR using the pCMV-Flag-Vpr plasmid as a template. A total of five Vpr mutants were generated, including Q65R, N41A, Q44A, E48A, and R88A.

For the TET2 degradation assay, HEK293T cells were grown to 80–90% confluence and then transfected with a mixture containing 1.5 μg of either wild-type or mutanted pCMV-Flag-Vpr plasmids (Q65R/N41A/Q44A/E48A/R88A), 1 μg of pCMV-TET2 (1129–1936)-Strep, and 12.5 μL of Lipofectamine 2000 (11668027, Invitrogen). For UNG2 degradation assay, HEK293T cells were transfected with a mixture containing 1.5 μg of either wild-type or mutant pCMV-Flag-Vpr plasmids (Q65R/R88A), 1 μg of wild-type or mutanted pCMV-UNG2-Strep plasmids (H124A/Y125A/T126A), and 12.5 μL of Lipofectamine 2000.

At 24 h post-transfection, cells were washed twice with ice-cold PBS and lysed on ice for 30 minutes using the lysis buffer (20 mM HEPES pH 7.5, 150 mM NaCl, 1% NP-40, 1 mM EDTA) supplemented with protease inhibitors. Lysates were clarified by centrifugation at 15,000 × g for 15 minutes at 4°C, and the supernatant was collected for western blotting analysis. Western blotting was performed with the following primary antibodies: mouse anti-Strep monoclonal antibody (S1002, Lanbolide) to detect TET2 and UNG2 proteins, and an HRP-conjugated anti-DYKDDDDK (Flag) mouse monoclonal antibody (F1105, Lanbolide) to detect Vpr expression. To ensure equal protein loading, β-actin was used as the loading control for TET degradation assays and was detected with an anti-β-actin mouse monoclonal antibody (A1101, Lanbolide), while Hsp90 was used as the loading control for UNG2 degradation assays and was detected with an anti-Hsp90 Rabbit monoclonal antibody (ZH5014, ZhongHe, China).

## Supporting information

S1 Fig Preparation and characterization of the complexes. **(A-C)** Biochemical characterization of the DDB1-VprBP complex in the absence and presence of Vpr-substrates. Size exclusion chromatography (SEC) elution profiles (left) and corresponding SDS-PAGE analysis (right) for **(A)** the DDB1-VprBP complex, (**B)** the DDB1-VprBP-Vpr-UNG2 quaternary complex, and **(C)** the DDB1-VprBP-Vpr-TET2 quaternary complex. In each SEC profile, the peak corresponding to the assembled complex is marked with an asterisk. **(D-F)** Representative negative staining EM micrographs of **(D)** the DDB1-VprBP complex, **(E)** the DDB1-VprBP-Vpr-UNG2 quaternary complex, and **(F)** the DDB1-VprBP-Vpr-TET2 quaternary complex. **(G-I)** Representative cryo-EM micrographs of **(G)** the DDB1-VprBP complex, **(H)** the DDB1-VprBP-Vpr-UNG2 quaternary complex, and **(I)** the DDB1-VprBP-Vpr-TET2 quaternary complex.
(TIF)

S2 Fig Cryo-EM data processing workflow of DDB1-VprBP. (TIF)

S3 Fig Cryo-EM data processing workflow of DDB1-VprBP-Vpr-UNG2. (TIF)

S4 Fig Cryo-EM data processing workflow of DDB1-VprBP-Vpr-TET2. (TIF)

S5 Fig Representative densities of the “ARM-up” DDB1-VprBP and DDB1-VprBP-Vpr-UNG2. (**A, B**) The representative densities of (**A**) the DDB1-VprBP complex in “ARM-up” state (presented at 3.8 σ) and (**B**) the DDB1-VprBP-Vpr-UNG2 complex (presented at 1.36 σ).
(TIF)

S6 Fig Local resolution maps, orientation distributions and Fourier shell coefficient (FSC) curves of the reconstructions. **(A-D)** The local resolution maps (left), orientation distribution diagrams (middle) and FSC curves (right) of **(A)** the DDB1-VprBP complex in “ARM-up” conformation, **(B)** the DDB1-VprBP complex in “ARM-down” conformation, **(C)** the DDB1-VprBP-Vpr-UNG2 complex, **(D)** the DDB1-VprBP-Vpr-TET2 complex. The local resolution maps were calculated using the software ResMap. The threshold used for measuring the resolution in the FSC curve is 0.143.
(TIF)

S7 Fig Structural prediction and analysis of full-length VprBP. (**A**) Schematic representation of the VprBP primary sequence, illustrating the boundaries for the ARM-like (yellow), LisH (orange), WD40 (blue), C-terminal Acidic (Magenta) domains as well as the unresolved regions in our experimental cryo-EM map (grey). (**B**) AlphaFold3-predicted structure of full-length VprBP, represented in ribbon representation and colored according to panel A. Regions corresponding to the unresolved density in our cryo-EM maps, including the long linkers within the ARM-like, LisH domain, and the Acidic domain, correlate with high intrinsic disorder segments in the predicted structure.
(TIF)

S8 Fig Position of the unresolved Acidic domain. Left, surface representation of the DDB1-VprBP complex with the ARM-like, WD40 domains and the partial Acidic domain colored yellow, blue, and magenta, respectively. Right, the zoomed-in view showing the potential pocket for accommodating the rest of the Acidic domain.
(TIF)

S9 Fig Electrostatic properties and structural conservation of the LisH dimer. **(A)** Electrostatic surface potential of the LisH dimer. The LisH dimer is shown as semi-transparent surface colored by electrostatic potential (red, negative; white, neutral; blue, positive). The charged residues at the C-terminal end of helix α4 are shown as sticks. **(B)** Structural comparison of LisH homologs. Structural superpositions of the VprBP LisH dimer (brown) with its homologous LisH dimers from TOPLESS-related protein 2 (TRP2) (light blue; PDB ID: 5C6Q), human SMU1 (pink; PDB ID: 6Q8F), and Lissencephaly-1 protein (Lis1) (light green; PDB ID: 1UUJ).
(TIF)

S10 Fig Comparison of “ARM-up” and “ARM-down” states and WD40 domain positioning in VprBP. Structural comparison of the “ARM-up” and “ARM-down” VprBP states. Ribbon diagram showing the superimposed “ARM-up” and “ARM-down” VprBP structures. Structures were aligned using the LisH and WD40 domains as a fixed reference. The “up” ARM-like domain and “down” ARM-like domain are colored yellow and cyan, respectively. The hinges connecting the ARM-like domain and LisH domain are highlighted in red (for the “up” state) and blue (for the “down” state), respectively.
(TIF)

S11 Fig Structural architecture and orientation of the VprBP WD40 domain. **(A)** Ribbon diagrams highlighting the H-box tail and β-propeller architecture of the WD40 domain. The β-propeller is constituted by seven WD40 repeats, each consisting of an antiparallel β-sheet formed by four short β-strands. **(B)** Spatial orientation of the WD40 domain. Ribbon diagram shows the spatial position of the WD40 domains (blue) relative to other domains of VprBP (gray). The red dashed lines indicate the pseudo-sevenfold axis of the WD40 domain.
(TIF)

S12 Fig Structural modeling of the CUL4A-Rbx1-DDB1-VprBP assembly across diverse BPB orientations. Structural models illustrate the recruitment of the CUL4A-Rbx1 catalytic module to the DDB1-BPB (green) in its different conformations. The model highlights a spatial relationship between the E2 recruiting protein Rbx1 (red) and the substrate binding domain VprBP-WD40 (blue). The dynamic orientation of the BPB domain results in a translation of the catalytic center relative to the substrate.
(TIF)

S13 Fig Structural basis of the Vpr-mediated UNG2 recruitment. **(A-B)** Comparison of the domain organizations in (**A**) the crystal structure (PDB ID: 5jk7) and (**B**) cryo-EM structure of DDB1-VprBP-Vpr-UNG2 complex. UNG2, VprBP-WD40, and DDB1-BPC are colored pink, blue, and cyan, respectively.
(TIF)

S14 Fig Structural and functional characterization of Vpr C-terminal region. (**A**) Density corresponding to Vpr C-terminus. A difference omit map shows the additional density near helix α2 of Vpr, which is almost parallel to helix α2 and has an average distance of 10 Å to the central axis of helix α2. (**B**) Functional validation of the Vpr C-terminal mutant. Western blot analysis showing that the Vpr C-terminal R88A mutant maintains its capacity to degrade UNG2. Western blots were performed in three independent biological replicates (*n* = 3) with one representative blot shown. Hsp90 was used as an internal loading control. Vpr mutant Q65R, which is deficient in VprBP binding, was used as a negative control. (**C)** Statistical quantification of biological replicates. Densitometric quantification derived from the three independent experimental repeats. Data are presented as mean. Error bars represent the standard deviation (SD). *n.s*., not statistically significant (*P* > 0.05); ***, *P* < 0.001.
(TIF)

S15 Fig Structural and functional characterization of VprBP-UNG2 interface. (**A**) Close contacts between UNG2 and VprBP ARM-like domain. Residues involved in close contacts (S2 Table) are highlighted in orange. (**B**) Functional validation of the VprBP-UNG2 interface. Western blot analysis showing that disrupting the contact interface of VprBP N-terminus and UNG2 abolishes Vpr-mediated UNG2 degradation. Western blots were performed in three independent biological replicates (*n* = 3) with one representative blot shown. Hsp90 was used as an internal loading control. (**C)** Statistical quantification of biological replicates. Densitometric quantification derived from the three independent experimental repeats. Data are presented as mean. Error bars represent the standard deviation (SD). **, *P* < 0.01.
(TIF)

S16 Fig Biochemical validation of interaction region between Vpr and TET2. (**A**) Schematic diagram showing the domain organization of TET2. (**B-C**) The TET2 disordered loop is essential for Vpr binding. The SEC elution profiles (top) and the corresponding SDS-PAGE analysis (bottom) are shown for (**B**) NusA-Vpr incubated with the TET2 catalytic domain lacking the internal loop (residues 1129–1936, Δ1481–1843) and (**C**) NusA-Vpr incubated with the intact TET2 catalytic domain (residues 1129–1936). The co-elution of Vpr and TET2 observed in (**C**) and the absence of co-elution in (**B**) demonstrates that the TET2 internal disordered loop is essential for the formation of a stable Vpr-TET2 complex. Asterisk indicates a contaminating protein.
(TIF)

S17 Fig The VprBP ARM-like domain adopts the “ARM-down” conformation in the DDB1-VprBP-Vpr-TET2 complex. (**A**) Comparison of the representative cryo-EM 2D class averages. Side-by-side comparison of 2D classes from the DDB1-VprBP-Vpr-UNG2 and DDB1-VprBP-Vpr-TET2 datasets, highlighting a distinct, additional density moiety at the base of the DDB1-VprBP-Vpr-TET2 complex. (**B**) Rigid-body fitting of the VprBP ARM-like domain. Structural docking of the ARM-like domain into the reconstructed map of the DDB1-VprBP-Vpr-TET2 complex. The densities corresponding to the ARM-like domain are colored yellow. (**C**) Comparison of the experimental densities of the two ARM-like domains (left and right) against the atomic model of the ARM-like domain (middle).
(TIF)

S18 Fig AlphaFold prediction and cellular validation of the interaction between TET2 and Vpr. **(A)** AlphaFold prediction of the Vpr-TET2 interaction. Residues involved in the interactions are shown as sticks. **(B)** Western blot analysis of TET2 co-transfected with Vpr or Vpr mutants in HEK293T cells. β-actin was used as an internal loading control. Vpr mutant Q65R, which is deficient in VprBP binding, was used as a negative control. (**C**) Statistical quantification of biological replicates. Densitometric quantification derived from the three independent experimental repeats. Data are presented as mean. Error bars represent the standard deviation (SD). *n.s.*, not statistically significant (*P* > 0.05); *, *P* < 0.05. (**D**) Functional validation of the Vpr C-terminal mutant in TET2 degradation. Western blot analysis showing that the Vpr C-terminal R88A mutant maintains its capacity to degrade TET2. Western blots were performed in three independent biological replicates (*n* = 3) with one representative blot shown. (**E**) Statistical quantification of biological replicates. Densitometric quantification derived from the three independent experimental repeats. Data are presented as mean. Error bars represent the standard deviation (SD). *n.s.*, not statistically significant (*P* > 0.05); *, *P* < 0.05; ***, *P* < 0.001.
(TIF)

S1 Table Data collection, 3D reconstruction and model statistics. (DOCX)

S2 Table Contact residues between UNG2 and the VprBP ARM-like domain. (DOCX)

S1 Video 3DFlex analysis of DDB1-VprBP-Vpr-TET2. (MP4)

S1 Raw Images Uncropped raw images of Coomassie-stained gels and Western blots. (A) Raw images of Coomassie-stained gels corresponding to S1A–C Fig. (B) Raw images of Western blots corresponding to S14B and S14C Fig. (C) Raw images of Western blots corresponding to S15B and S15C Fig. (D) Raw images of Western blots corresponding to S18B and S18C Fig. (E) Raw images of Western blots corresponding to S18D and S18E Fig.
(PDF)
