# Supplementary material for: Structural basis of diverse substrate recruitment by the HIV-1 Vpr-hijacked CRL4(VprBP) E3 ligase
Source: PLoS Pathog. 2026 Oct 1;22(10):e1014610. doi: 10.1371/journal.ppat.1014610 (PMC13630203; doi:10.1371/journal.ppat.1014610)
Supplement: Supplementary file 1 [file ppat.1014610.s022.pdf]

A

S1A Fig

The SDS-PAGE gel after the size exlusion chromotography

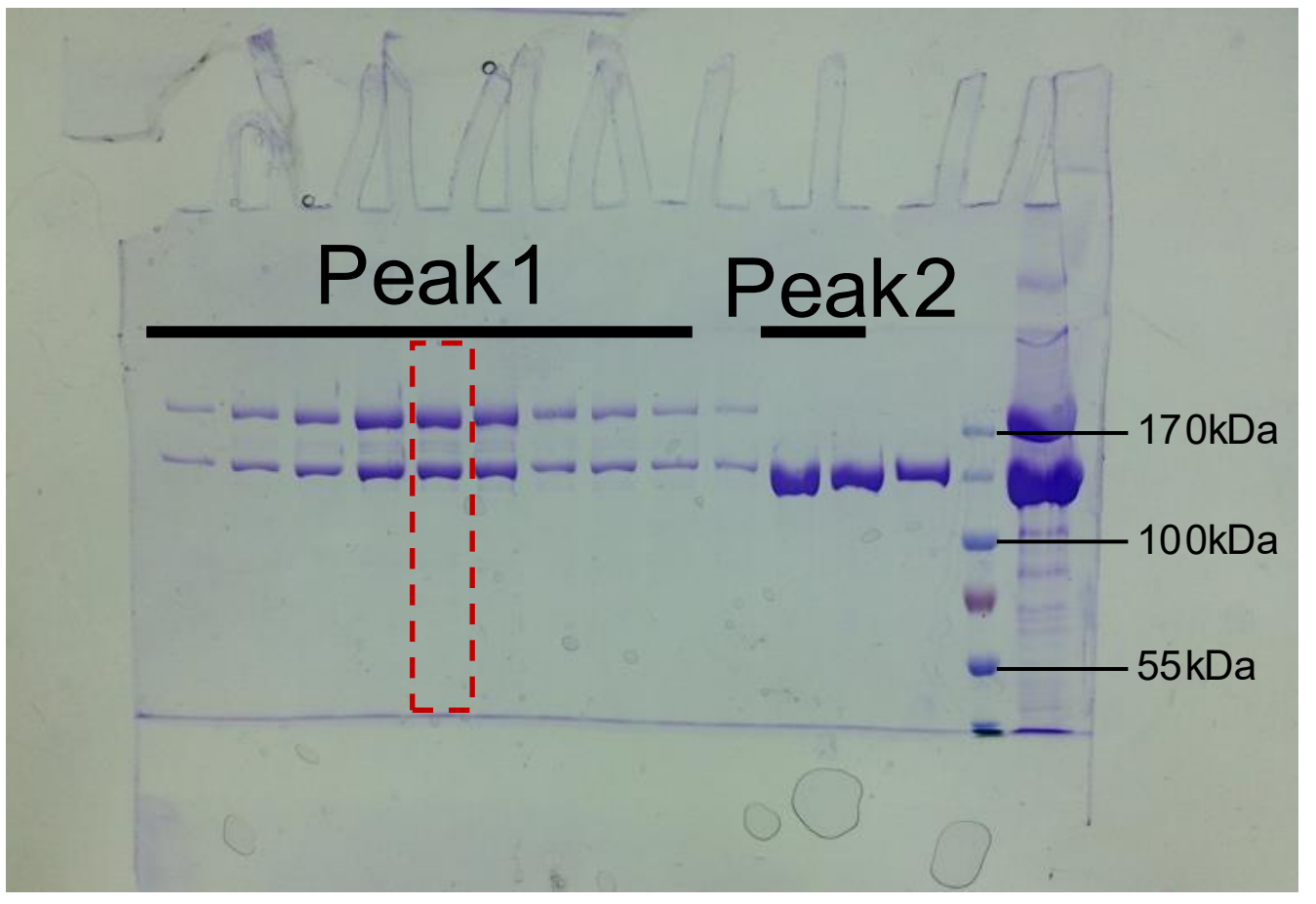

S1B Fig

The SDS-PAGE gel after the size exlusion chromotography

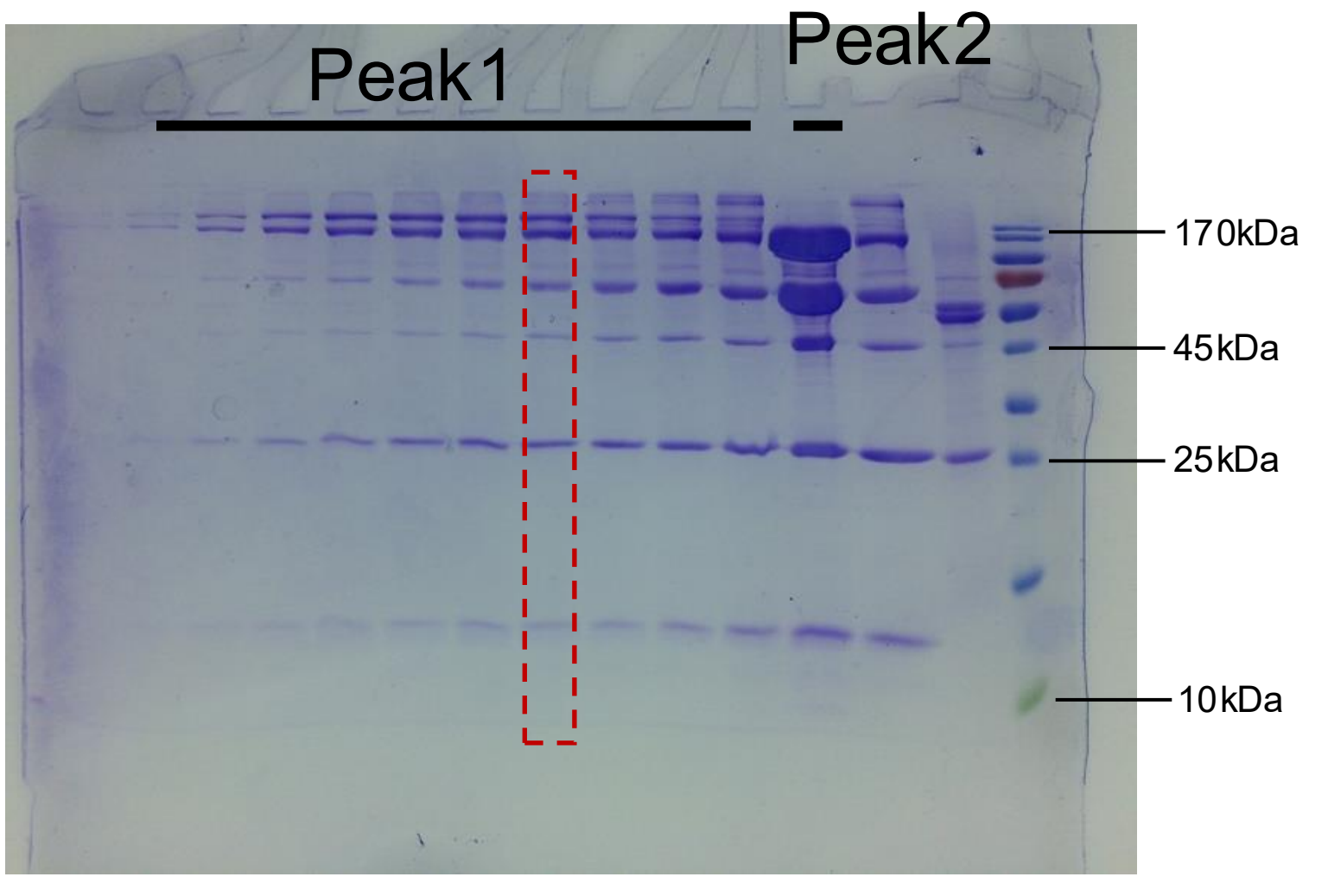

S1C Fig

The SDS-PAGE gel after the size exlusion chromotography

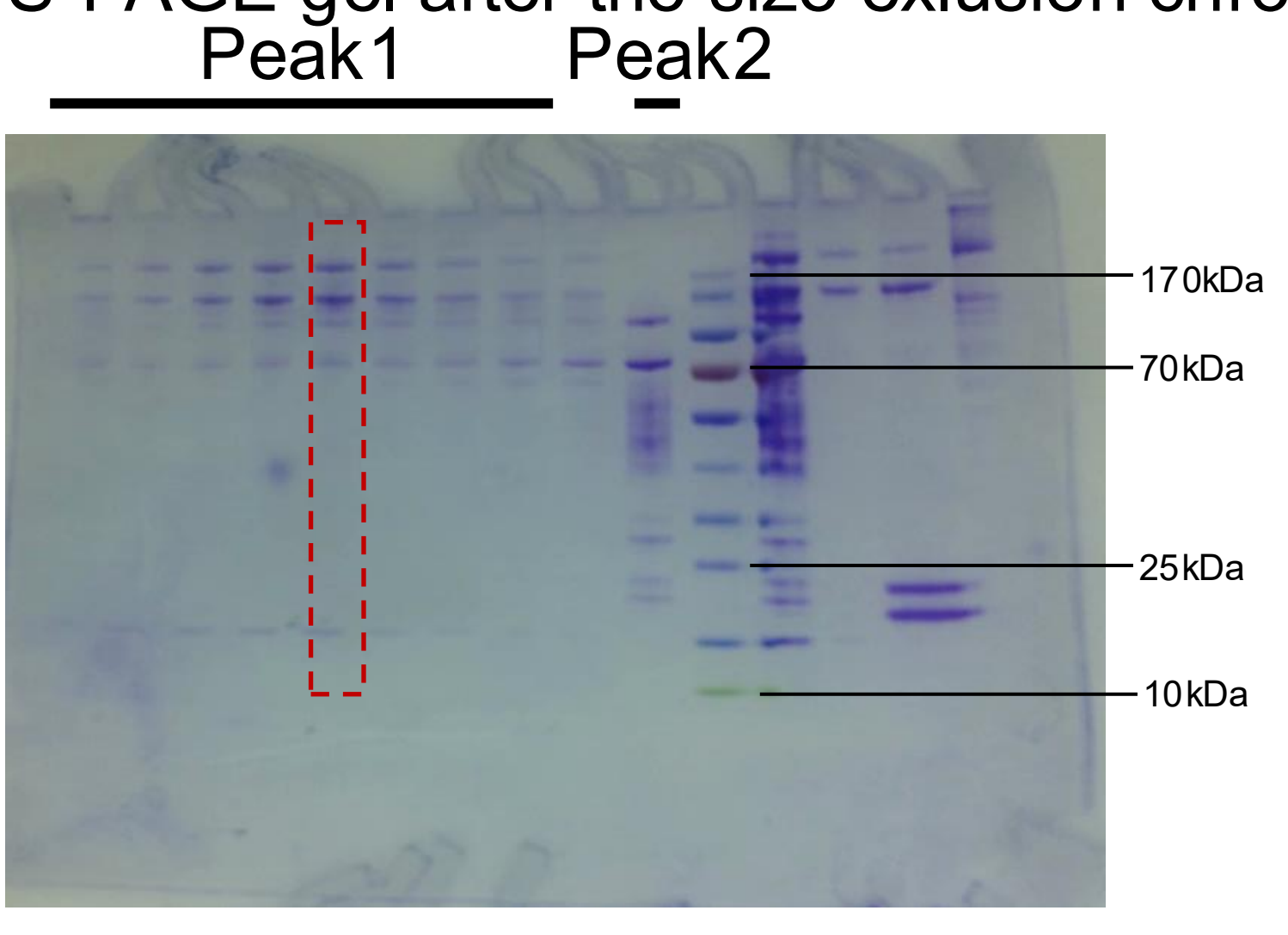

B

S14B, C Fig

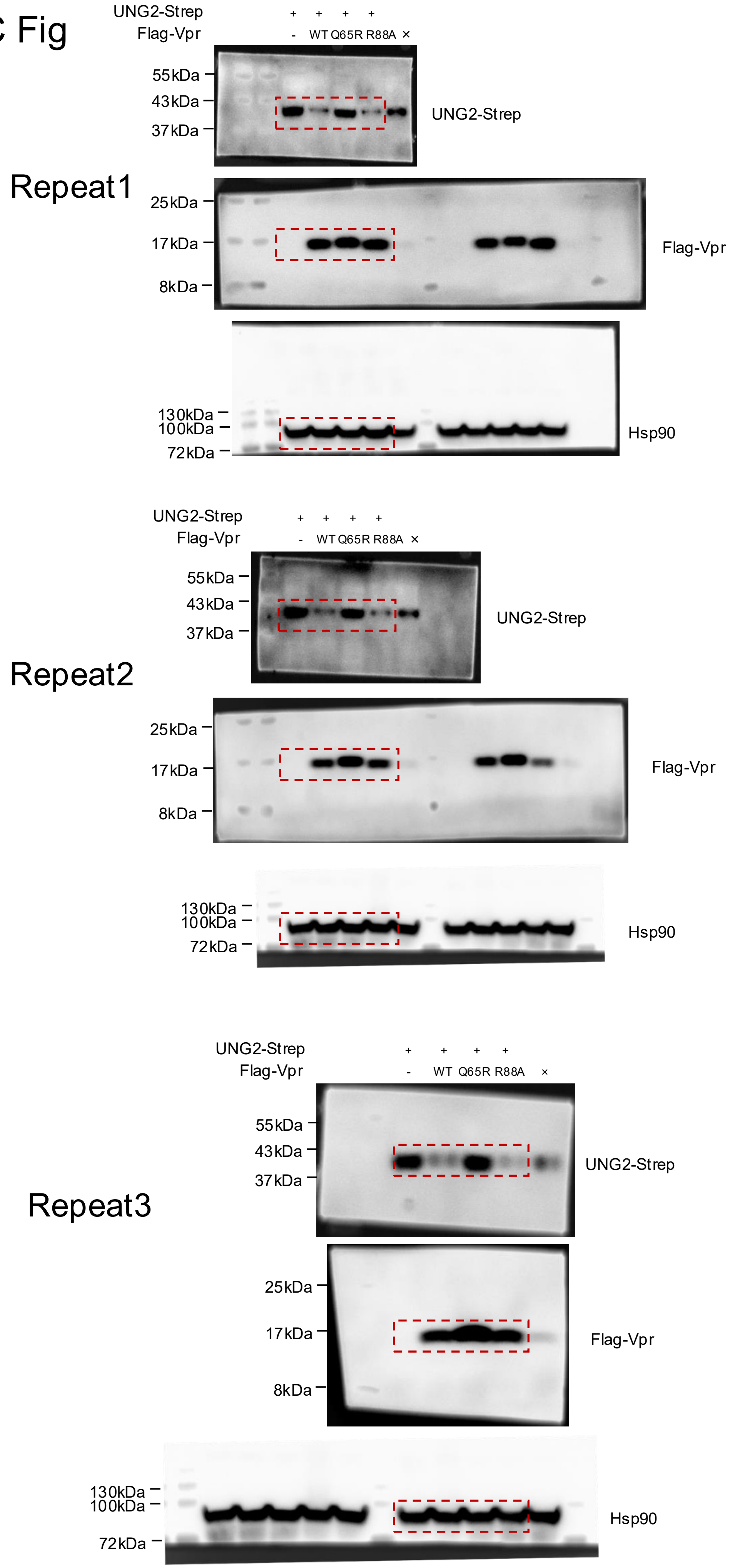
UNG2-Strep + + + +
Flag-Vpr - WT Q65R R88A ×
55kDa
43kDa
37kDa
UNG2-Strep
Repeat1
25kDa
17kDa
8kDa
Flag-Vpr
130kDa
100kDa
72kDa
Hsp90
UNG2-Strep + + + +
Flag-Vpr - WT Q65R R88A ×
55kDa
43kDa
37kDa
UNG2-Strep
Repeat2
25kDa
17kDa
8kDa
Flag-Vpr
130kDa
100kDa
72kDa
Hsp90
UNG2-Strep + + + +
Flag-Vpr - WT Q65R R88A ×
55kDa
43kDa
37kDa
UNG2-Strep
Repeat3
25kDa
17kDa
8kDa
Flag-Vpr
130kDa
100kDa
72kDa
Hsp90

C S15B, C Fig

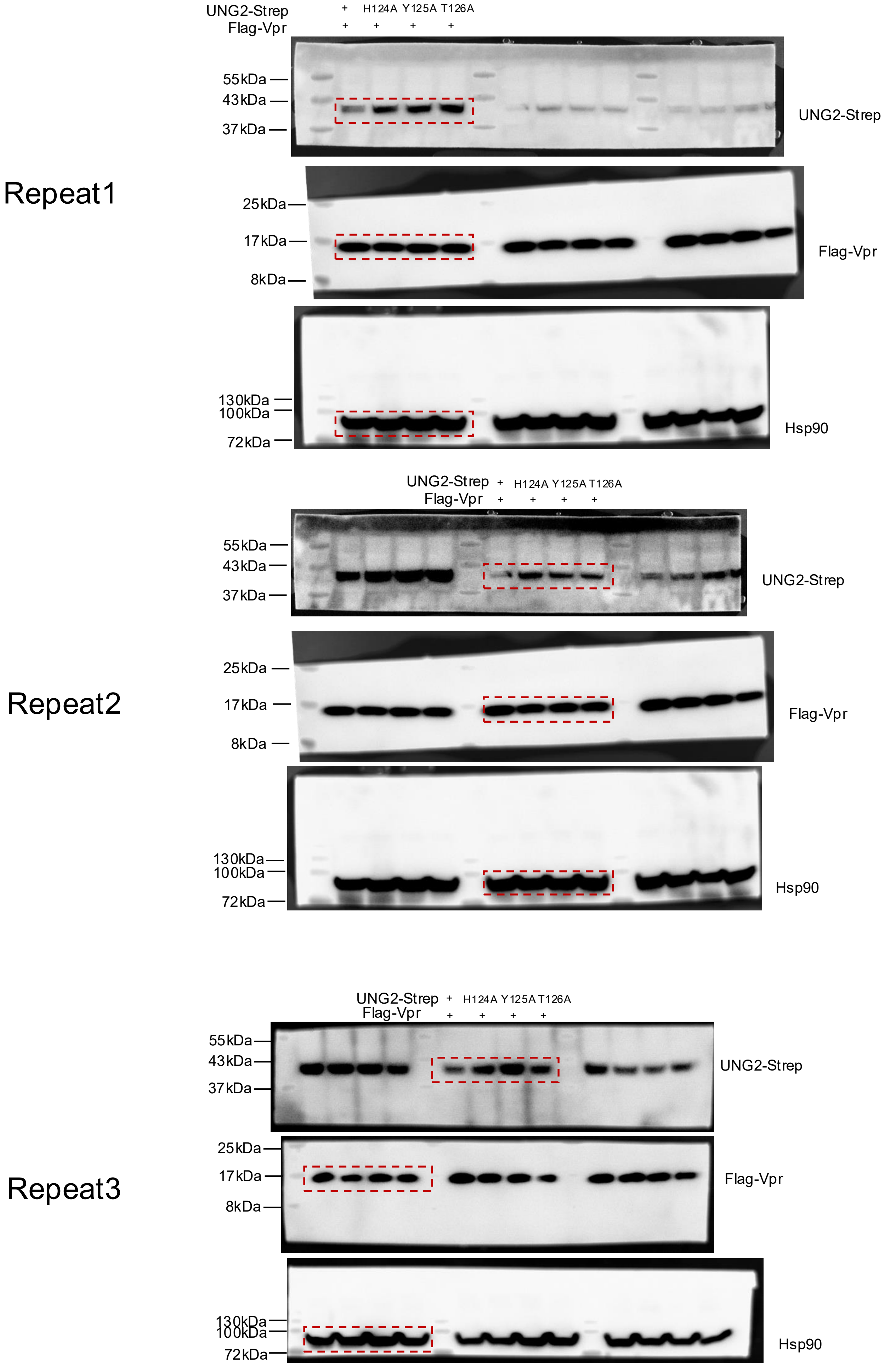
UNG2-Strep
Flag-Vpr
+ H124A Y125A T126A
+ + + +
55kDa
43kDa
37kDa
UNG2-Strep
Repeat1
25kDa
17kDa
8kDa
Flag-Vpr
130kDa
100kDa
72kDa
Hsp90
UNG2-Strep
Flag-Vpr
+ H124A Y125A T126A
+ + + +
55kDa
43kDa
37kDa
UNG2-Strep
Repeat2
25kDa
17kDa
8kDa
Flag-Vpr
130kDa
100kDa
72kDa
Hsp90
UNG2-Strep
Flag-Vpr
+ H124A Y125A T126A
+ + + +
55kDa
43kDa
37kDa
UNG2-Strep
Repeat3
25kDa
17kDa
8kDa
Flag-Vpr
130kDa
100kDa
72kDa
Hsp90

D S18B, C Fig

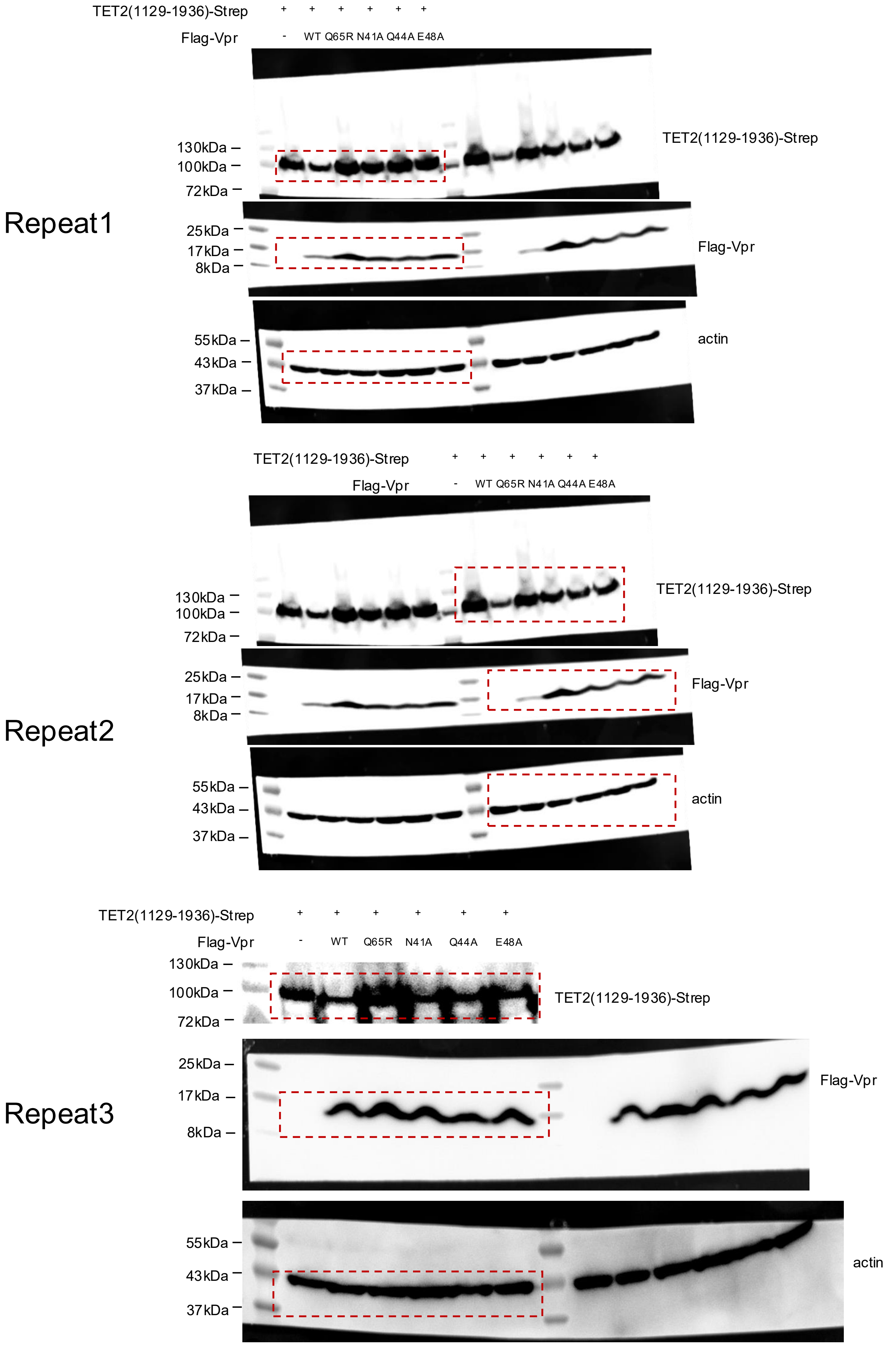
TET2(1129-1936)-Strep + + + + + +
Flag-Vpr - WT Q65R N41A Q44A E48A
130kDa
100kDa
72kDa
TET2(1129-1936)-Strep
Repeat1
25kDa
17kDa
8kDa
Flag-Vpr
55kDa
43kDa
37kDa
actin
TET2(1129-1936)-Strep + + + + + +
Flag-Vpr - WT Q65R N41A Q44A E48A
130kDa
100kDa
72kDa
TET2(1129-1936)-Strep
25kDa
17kDa
8kDa
Flag-Vpr
Repeat2
55kDa
43kDa
37kDa
actin
TET2(1129-1936)-Strep + + + + + +
Flag-Vpr - WT Q65R N41A Q44A E48A
130kDa
100kDa
72kDa
TET2(1129-1936)-Strep
25kDa
17kDa
8kDa
Flag-Vpr
Repeat3
55kDa
43kDa
37kDa
actin

E S18D, E Fig

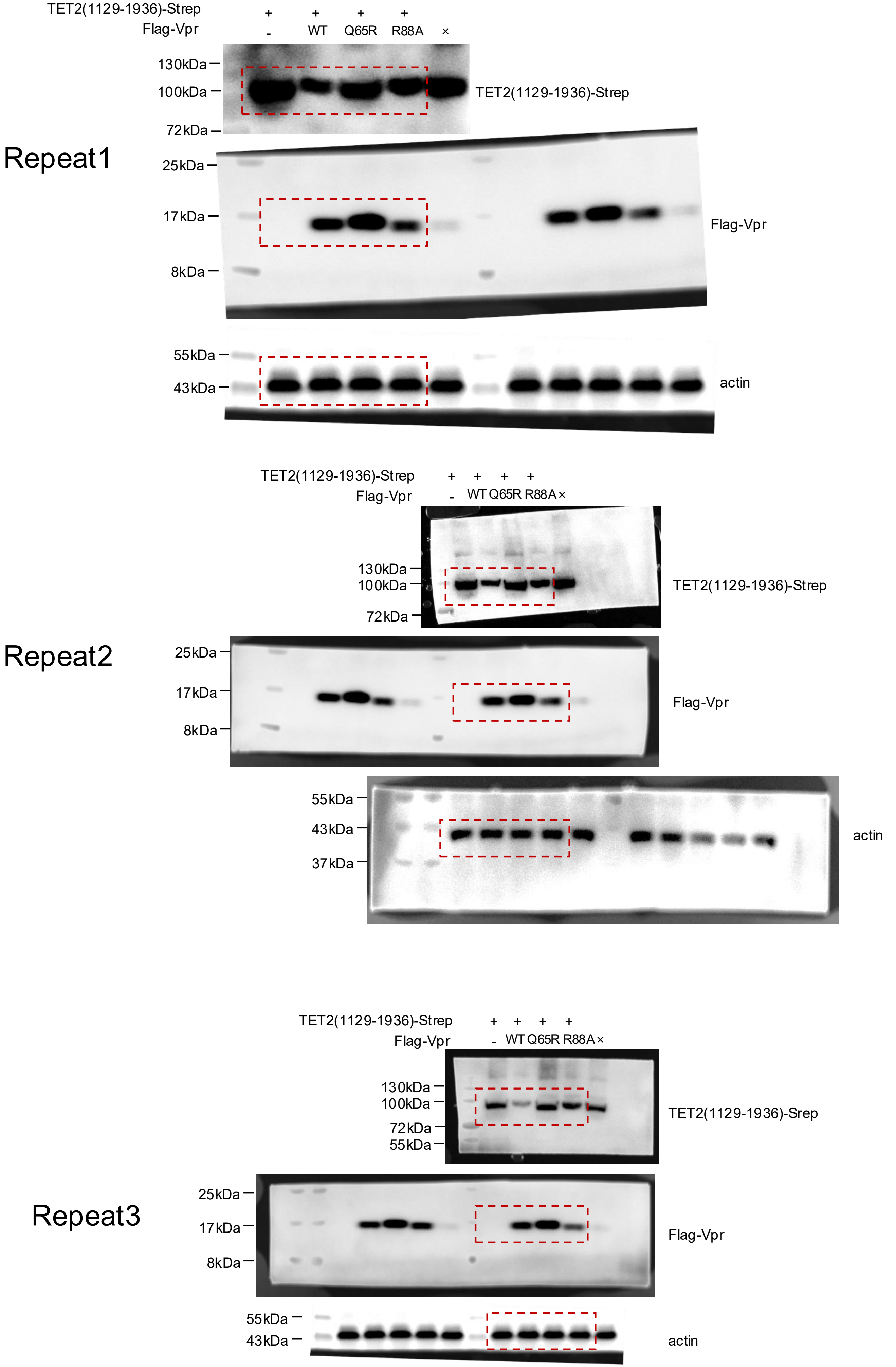
TET2(1129-1936)-Strep
+ + + +
Flag-Vpr
- WT Q65R R88A ×
130kDa
100kDa
72kDa
TET2(1129-1936)-Strep
Repeat1
25kDa
17kDa
8kDa
Flag-Vpr
55kDa
43kDa
actin
TET2(1129-1936)-Strep
+ + + +
Flag-Vpr
- WT Q65R R88A ×
130kDa
100kDa
72kDa
TET2(1129-1936)-Strep
Repeat2
25kDa
17kDa
8kDa
Flag-Vpr
55kDa
43kDa
37kDa
actin
TET2(1129-1936)-Strep
+ + + +
Flag-Vpr
- WT Q65R R88A ×
130kDa
100kDa
72kDa
55kDa
TET2(1129-1936)-Srep
Repeat3
25kDa
17kDa
8kDa
Flag-Vpr
55kDa
43kDa
actin
