# Supplementary material for: Structural basis of diverse substrate recruitment by the HIV-1 Vpr-hijacked CRL4(VprBP) E3 ligase
Source: PLoS Pathog. 2026 Oct 1;22(10):e1014610. doi: 10.1371/journal.ppat.1014610 (PMC13630203; doi:10.1371/journal.ppat.1014610)
Supplement: Supplementary file 2 [file ppat.1014610.s019.docx]

**S1 Table. Data collection, 3D reconstruction and model statistics.**

|  | **DDB1-VprBP “ARM-up” state**  **EMD-31765**  **PDB ID:7V7B** | **DDB1-VprBP “ARM-down” state**  **EMD-58935** | **DDB1-VprBP-Vpr-UNG2(94-313)**  **EMD-31766**  **PDB ID: 7V7C** | **DDB1-VprBP-Vpr-TET2(1129-1936)**  **EMD-58936** |
| --- | --- | --- | --- | --- |
| **Data collection** |  | | | |
| Micrograph | 2194 | 2194 | 3995 | 4903 |
| Voltage (kV) | 300 | 300 | 300 | 300 |
| Detector | K2 camera | K2 camera | K3 camera | K3 camera |
| Pixel Sixe (Å/pixel) | 1.091 | 1.091 | 1.0742 | 1.25 |
| Defocus range (µm) | 1.0-3.0 | 1.0-3.0 | 1.0-3.0 | 1.0-3.0 |
| Electron  Exposure (e^-^/Å^2^) | 50 | 50 | 50 | 50 |
| **Reconstruction** |  | | | |
| Software | RELION | RELION | RELION | cryoSPARC |
| Final particles (No.) | 84405 | 127356 | 145568 | 144145 |
| Resolution (Å) | 4.2 | 9.6 | 3.7 | 7.7 |
| Symmetry imposed | C2 | C2 | C2 | C1 |
| FSC threshold | 0.143 | 0.143 | 0.143 | 0.143 |
| Average B-factor (Å^2^) | -192 | -200 | -236 | -300 |
| **Model building** |  | | | |
| Software | Coot |  | Coot |  |
| Model composition |  | | | |
| Residues | 4484 |  | 5084 |  |
| Non-hydrogen atoms | 35366 |  | 40264 |  |
| **Refinement** |  | | | |
| Software | Phenix & RosettaCM |  | Phenix & RosettaCM |  |
| R.M.S deviations |  |  |  |  |
| Bond length (Å) | 0.0051 |  | 0.0057 |  |
| Bond angles (°) | 1.18 |  | 1.18 |  |
| Validation |  |  |  |  |
| MolProbity score | 1.84 |  | 1.83 |  |
| Clashscore | 5.8 |  | 5.68 |  |
| Rotamer outlier (%) | 0 |  | 0.09 |  |
| Ramachandran  Plot statistics |  |  |  |  |
| Preferred (%) | 90.02 |  | 90.96 |  |
| Allowed (%) | 9.94 |  | 9.00 |  |
| Outlier (%) | 0.04 |  | 0.04 |  |
