# Supplementary material for: Structural basis of diverse substrate recruitment by the HIV-1 Vpr-hijacked CRL4(VprBP) E3 ligase
Source: PLoS Pathog. 2026 Oct 1;22(10):e1014610. doi: 10.1371/journal.ppat.1014610 (PMC13630203; doi:10.1371/journal.ppat.1014610)
Supplement: Supplementary file 3 [file ppat.1014610.s020.docx]

**S2 Table. Contact residues between UNG2 and the VprBP ARM-like domain.**

| **UNG2 Residues** | **VprBP Residues** | **Distance (Å)** |
| --- | --- | --- |
| Thr126 | Asp82 | 2.46 |
| Tyr125 | Asp82 | 3.17 |
| His124 | Lys80 | 3.22 |
| Tyr125 | Lys80 | 3.60 |
